# Interfacial Roughness‐Reinforced Magnetic Nanosheet Colloidal Gels for Stable Embolization and Magnetothermal Therapy of Hepatic Tumor

**DOI:** 10.1002/advs.202507096

**Published:** 2025-08-13

**Authors:** Xingyu Liu, Sheng Chen, Yonghong Song, Bing Chen, Xu Yan, Rui Qiu, Jinlong Hu, Baoqiang Cao, Hanye Xing, Tao Zhou, Liang Dong, Yang Lu

**Affiliations:** ^1^ Key Laboratory of Value‐Added Catalytic Conversion and Reaction Engineering School of Chemistry and Chemical Engineering Hefei University of Technology Hefei Anhui 230009 P. R. China; ^2^ Hangzhou Institute of Medicine (HIM) Chinese Academy of Sciences Zhejiang Cancer Hospital Hangzhou Zhejiang 310018 P. R. China; ^3^ Department of General Surgery Department of Ultrasonics Department of Interventional Radiology Anhui No. 2 Provincial People's Hospital Hefei Anhui 230041 P. R. China

**Keywords:** embolization therapy, interfacial design, magnetic colloidal gel, roughness, specific surface area

## Abstract

Injectable hydrogels have been developed for transcatheter arterial embolization (TAE) of hepatic tumor owing to their flexibility in injection and embolization. However, the dual requirement for high mechanical robustness and good injectability poses a fundamental challenge, as improvements in one often compromise the other. Colloidal gels offer tunable mechanical properties and feasible injectability through the bottom‐up assembly of building blocks. Here an interfacial reinforcement strategy is developed by enhancing specific surface area and roughness to fabricate nanosheet‐mica (NM) magnetic colloidal gels (NMMG). The negatively charged NM, exfoliated from natural ground mica (GM), possessed increased binding sites, while in situ grown Fe_3_O_4_ nanoparticles enhanced building block roughness, reducing intergranular displacement and thereby maintaining mechanical strength while ensuring injectability. Compared with GM‐based magnetic colloidal gels, NMMG can be injected through 2.6 F catheter with ≈20 N force (40% of manual limit), while storage modulus improved from ≈800 to ≈1800 Pa (2.25‐fold higher than embolization minimum limit). Transcatheter embolization with NMMG significantly inhibited tumor growth. Furthermore, NMMG‐mediated minimally invasive therapies, including TAE combined with magnetothermal therapy, achieved synergistic therapeutic effects in hepatic tumor‐bearing rabbits. The rough‐interface strengthening strategy facilitates the development of high‐performance colloidal gels with mechanical integrity and tailored functions.

## Graphical abstract

A rough‐interface reinforcement strategy is implemented to construct nanosheet‐mica‐based magnetic colloidal hydrogels with enhanced mechanical robustness and injectability. These hydrogels achieve stable vascular embolization and effective magnetothermal performance, offering synergistic therapeutic benefits in hepatic tumor treatment.

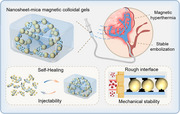

## 1 Introduction

Injectable hydrogels have received great attention in the hepatocellular carcinoma (HCC) treatment recently,^[^ ^1^, ^2^, ^3^ ^]^ ascribing to self‐healing,^[^ ^4^ ^]^ and adjustable mechanical properties,^[^ ^5^ ^]^ especially for minimally invasive administration such as transcatheter arterial embolization (TAE),^[^ ^6^, ^7^, ^8^ ^]^ which is the preferred option for treating middle‐ and advanced‐stage HCC.^[^ ^9^, ^10^, ^11^ ^]^ Moreover, as commonly used TAE agents, the microspheres or Lipiodol, are limited by the recanalization or nonspecific embolization.^[^ ^12^, ^13^ ^]^ Injectable hydrogels display the advantages that could be delivered using clinically relevant catheter and form a blocking completely in various vessel geometries and sizes, avoiding embolization failure and recurrent bleeding.^[^ ^14^, ^15^, ^16^, ^17^ ^]^ In addition, functional hydrogels offer the flexibility to deliver combination therapeutics, since single TAE treatment is inefficient to restrict the rapid growth of HCC, other therapies are usually synergistically applied with TAE to improve the efficacy.^[^ ^18^, ^19^, ^20^, ^21^ ^]^ Notably, the TAE process of the injected hydrogel can be divided into two stages, first, the hydrogels are injected into the targeted blood vessel through the medical syringe with the injected force lower than 50 N, which is considered as tolerable limit.^[^ ^22^, ^23^ ^]^ Second, the hydrogel must remain the stable effect and resist the pressure of blood to block the vessel, which the storage modulus (G′) reported should be higher than 800 Pa.^[^ ^6^ ^]^ Given that the shear stress overcoming the mechanical stability of the network and causing the hydrogel to break down into an injectable flowing state, there is an imbalance to fulfill the requirements of the hydrogels in terms of the injectability through catheters and the mechanical strength.^[^ ^24^, ^25^, ^26^ ^]^ Consequently, there is still a big challenge to achieve the tailored hydrogel with feasible injected force and stable mechanical properties enabling personalized and precise TAE treatments.

Colloidal gels (CGs), as a variant of hydrogels, exhibit excellent shear‐thinning and self‐healing performance contributing to self‐assembled colloidal particles and inter‐particle bonds.^[^ ^27^, ^28^, ^29^ ^]^ With the bottom‐up assembly of building blocks, hierarchical design is allowed by modifying the particles surface and combining them as modular biomaterial platforms.^[^ ^30^, ^31^, ^32^, ^33^, ^34^ ^]^ Moreover, owing to the physicochemical features of self‐assembled blocks within CGs, the building blocks’ characteristics that constitute the networks of CGs offer selectivity to enhance properties.^[^ ^35^, ^36^, ^37^ ^]^ Despite CGs being especially excellent in injectability, weakness in mechanical properties restrains their practical applications.^[^ ^31^, ^35^ ^]^ To this end, the influence of particle morphology (such as dimensions,^[^ ^30^, ^38^, ^39^ ^]^ surface roughness,^[^ ^40^ ^]^ stiffness^[^ ^41^ ^]^ and adhesion^[^ ^42^ ^]^) on the self‐assembly and fractal structures of colloidal aggregates has garnered extensive attention in the regulation of CGs’ properties by controllable interfacial interactions,^[^ ^43^, ^44^ ^]^ which can circumvent the pitfalls of current CGs without the need of rheological additives. For instance, imparting surface roughness to the primary particles as building blocks increased the toughness of CGs by an order of magnitude.^[^ ^40^ ^]^ Therefore, designing interfaces with an effective increase of cross‐linking sites between the building blocks can enhance mechanical properties but also maintain injectability, which is meaningful to constructing CGs embolic agents for promising clinical applications.

**Figure 1:**
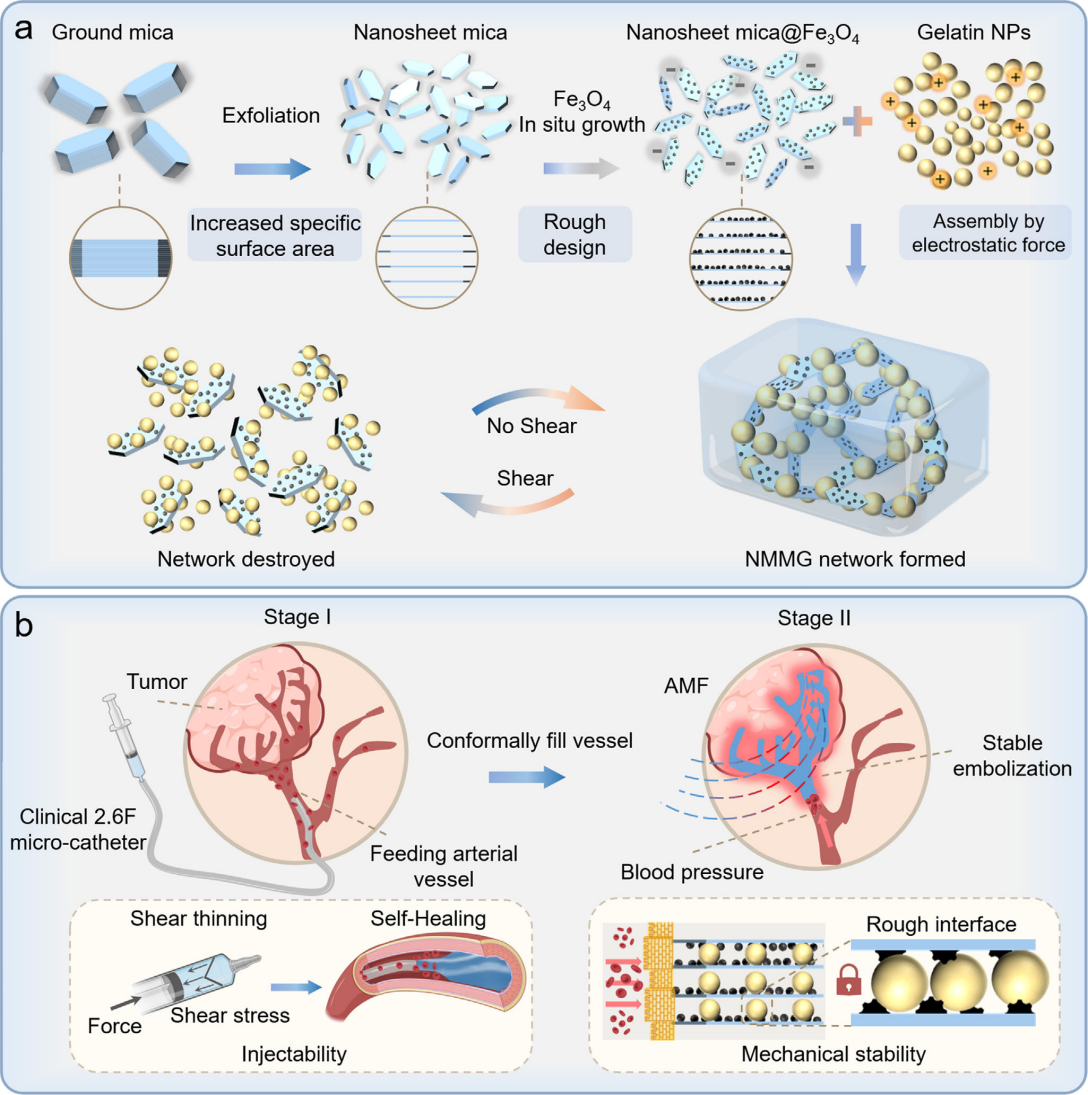
Schematic illustration of an interfacial reinforcement strategy to design injectable and mechanically stable nanosheetmica magnetic colloidal gels (NMMG) by increasing the specific surface area and roughness of building blocks. a) The schematic preparation processes of building blocks with enhanced interfacial roughness and their assembly into NMMG. b) Rough interfaces strengthen the mechanical stability between building blocks, enabling NMMG to achieve stable tumor embolization through a 2.6 F catheter and enhancing the therapeutic effect of TAE combined with MHT.

2D materials, contributing to the high specific surface area, high aspect ratio, possess a high number of surface anchoring sites to increase the crosslinking degree among the interface.^[^ ^45^, ^46^, ^47^, ^48^, ^49^ ^]^ As a natural 2D non‐metallic clay, mica shows the characteristics such as biocompatibility, abundance and mechanical properties, has been widely used in biomedicine, inorganic filler, device and other fields.^[^ ^50^, ^51^, ^52^, ^53^, ^54^ ^]^ Especially, in our previous work,^[^ ^50^ ^]^ the layered structure of ground mica (GM) can be effectively exfoliated into nanosheets‐mica (NM) with a large scale, exhibiting excellent radius‐thickness ratio with a highly increased specific surface area. Herein, as shown in **Figure** **1**, the Fe_3_O_4_ nanoparticles were in situ grown on the surface of micas with enhanced roughness and magnetic heating effect under alternating magnetic field (AMF). We reported an interfacial reinforcement strategy in the design of injectable and mechanically stable CG via enhanced specific surface area and roughness of building blocks. Compared with CG of the GM@Fe_3_O_4_ as building blocks assembled with Gelatin NPs through electrostatic force (GM magnetic colloidal gel, GMMG), the NM@Fe_3_O_4_ and Gelatin NPs assembled colloidal gel (NM magnetic colloidal gel, NMMG) has better injection performance with an injection force of ≈20 N and more than twofold increased mechanical performance with stable network structure. Simultaneously, contributed to the enhanced specific surface area resulted more Fe_3_O_4_ growth, NMMG possessed a more efficient magnetothermal effect compared to GMMG. Furthermore, the NMMG realized efficient therapeutic on tumor‐bearing rabbit model through minimally invasive therapies including ultrasound guided interventional magnetic hyperthermia therapy (MHT) and the incorporation of TAE and MHT.

## 2 Results and Discussion

### 2.1 Preparation and Characterization of Binary Composite Colloidal Gels

**Figure 2:**
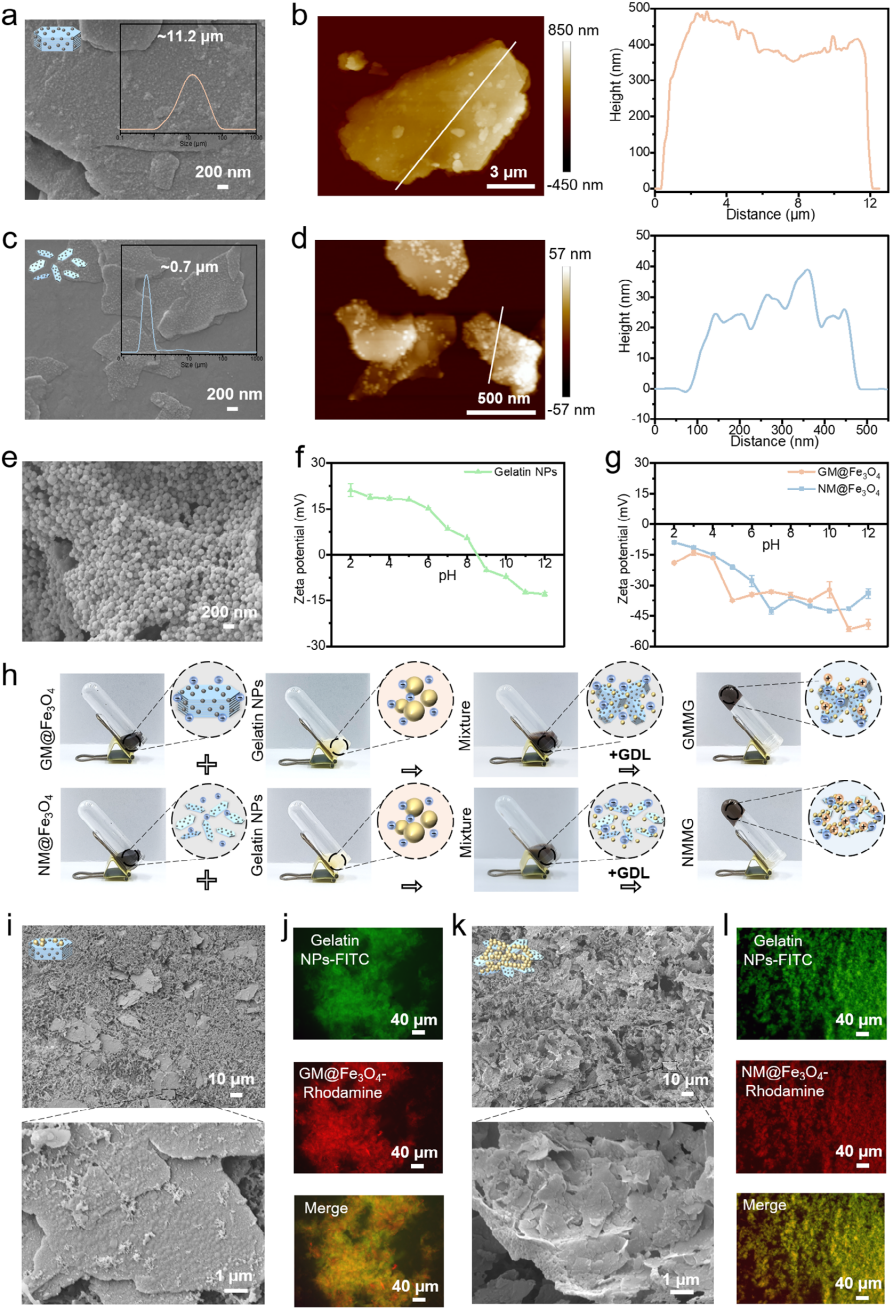
Preparation and observation of CGs. a,c) SEM and b,d) AFM images of GM@Fe_3_O_4_ and NM@Fe_3_O_4_, respectively. Size distributions were inserted in the right of (a, c); right curves in (b, d) displayed the thickness of GM@Fe_3_O_4_ and NM@Fe_3_O_4_. e) SEM of Gelatin NPs. f) Zeta potentials of Gelatin NPs, g) GM@Fe_3_O_4_ and NM@Fe_3_O_4_ in various pH values. h) The schematic illustration of the preparation processes of GMMG and NMMG. i,k) The SEM observation of network structure within GMMG and NMMG, respectively. Representative fluorescence microscopy images of assembled building blocks in the network of j) GMMG and l) NMMG.

To prepare 2D building blocks for CGs, natural GM was first exfoliated into mica nanosheets according to the method described in previous work.^[^ ^50^ ^]^ After exfoliation, The TEM images of NM showed a 2D structure with an average diameter of ≈ 0.65 µm, which was much smaller than the average diameter (≈ 10.62 µm) of GM (Figure S1a,c, Supporting Information). Further analysis by atomic force microscope (AFM) showed a similarly intense change in thickness that the height of GM decreased from ≈ 300 to ≈ 1 nm of NM (Figure S2, Supporting Information), which indicated the high radius‐to‐thickness ratio. Then, ≈17 nm Fe_3_O_4_ nanoparticles were grown in situ on the surface of GM or NM, yielding GM@Fe_3_O_4_ and NM@Fe_3_O_4_, respectively (Figure S3, Supporting Information). As shown in **Figure** **2**a,c, the nanoparticles can be visually attached to the surface of both GM and NM, and their diameter exhibited no significant increase after the in situ growth (inset in Figure 2a,c). Subsequently, the TEM images (Figure S1b,d, Supporting Information) and X‐ray diffraction (XRD) patterns also provided evidence for successful Fe_3_O_4_ growth (Figure S4, Supporting Information).^[^ ^50^, ^55^, ^56^, ^57^ ^]^ In the XPS spectrum, the peaks appearing at 711 and 724 eV corresponded to the Fe 2p_3/2_ and Fe 2p_1/2_ binding energies, respectively, indicative of the presence of iron oxide nanoparticles (Figure S5, Supporting Information).^[^ ^56^, ^57^ ^]^ After the successful growth of Fe_3_O_4_, there is no significant alteration in the height of GM@Fe_3_O_4_ (Figure 2b), whereas the height of NM@Fe_3_O_4_ increased to ≈16 nm (Figure 2d). The building blocks of Gelatin NPs were spherical (Figure 2e) with a hydrodynamic diameter of ≈300 nm (Figure S6, Supporting Information). The Gelatin NPs showed charge inversion characteristic with an isoelectric point of 8–9 via the regulation of pH values (Figure 2f). The zeta potential of GM@Fe_3_O_4_ and NM@Fe_3_O_4_ still maintained a negative charge under different pH values (Figure 2g), which provided the feasibility of electrostatic assembly between the Gelatin NPs and magnetic mica composites. As shown in Figure 2h, when the Gelatin NPs and NM@Fe_3_O_4_ were mixed in an alkaline environment, the binary mixture maintained a fluid‐like state, then the pH regulation mediated sol‐to‐gel transformation visually exhibited the process of hydrogel formation by electrostatic assembly due to the whole system pH going below the isoelectric point of Gelatin NPs. The microstructures of GMMG (Figure 2i) presented Gelatin NPs formed porous colloidal gel network instead of the uniform aggregates of GM@Fe_3_O_4_ and Gelatin NPs, where GM@Fe_3_O_4_ doped inhomogeneously and greatly impeded the continuous network of nanoparticles. By contrast, from the SEM images of NMMG (Figure 2k), the NM@Fe_3_O_4_ and Gelatin NPs assembled tightly and connected to form a homogeneous structure in a colloidal network. Furthermore, the Gelatin NPs and mica composites were dyed by FITC and Rhodamine, respectively. The fluorescent images of GMMG (Figure 2j) showed an inhomogeneous network structure with a low overlap ratio of the two colors. By contrast, the fluorescent images of NMMG (Figure 2l) visually demonstrated that the connected aggregates had a uniformly distributed structure with almost exact superposition of the two colors. Compared with GM@Fe_3_O_4_, NM@Fe_3_O_4_ formed CGs exhibited better network structure continuity.

### 2.2 Electrostatic Interaction Regulation between Building Blocks

**Figure 3:**
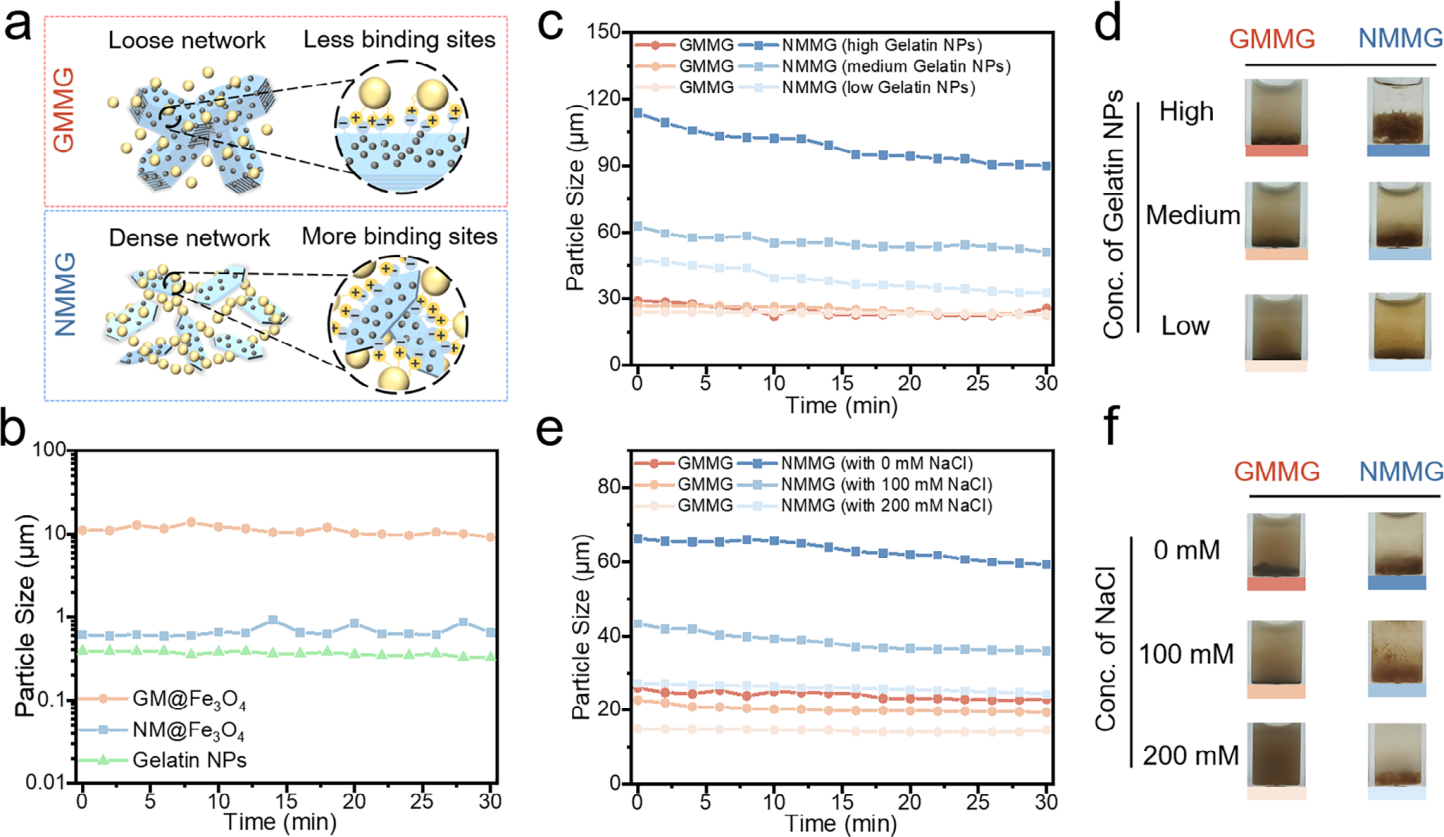
a) Schematic depiction of the crosslinking nodes between assembled building blocks in the network formation. b) Particle size of building blocks monitored by DLS. c,d) The size and representative photographs of colloidal clusters containing different contents of positively charged Gelatin NPs with the same content in negative building blocks. e,f) The size and representative photographs of colloidal clusters assembled by the same building blocks concentration and mixed in aqueous solutions containing 0, 100 or 200 mM NaCl.

As illustrated in **Figure** **3**a, compared with GMMG, it could be speculated that the NMMG possessed a denser network owing to the more electrostatic interaction binding sites. The electrostatic interaction between Gelatin NPs and mica composites mediated aggregates was monitored by DLS analysis.^[^ ^58^ ^]^ Moreover, based on the monitoring of building blocks separately (Figure 3b), their particles’ size remained essentially unchanged for 30 min, indicating that the aggregation in the GMMG or NMMG suspension was assembled by electrostatic interaction rather than settlement of particle aggregation. The mixtures of different contents in Gelatin NPs with the same content in negative building blocks were carried out to form diverse colloidal clusters (Figure 3c). It's noteworthy that the increased particle size of aggregation in the NMMG suspension appeared concentration‐dependent behavior with the increased addition of Gelatin NPs. By contrast, GMMG showed no obvious change in particle size. Such difference demonstrated the increased contact area between the building blocks in NMMG. Meanwhile, the corresponding images of various suspensions (Figure 3d) further visually confirmed more stronger attraction between Gelatin NPs and NM@Fe_3_O_4_. With the sizes of precipitate increased considerably, the supernatants gradually became clarified with the increased content of Gelatin NPs.

Recent studies have reported that the ionic strength in solution has a significant shielding effect on the electrostatic attraction.^[^ ^59^, ^60^ ^]^ Upon dispersing building blocks into NaCl solutions, both aggregates revealed decreased size as the NaCl concentration increased (Figure 3e), confirming that electrostatic interactions were the main driving force for colloidal assembly. Combined with the photos of Figure 3f, the GMMG with 200 mm NaCl showed a particle size close to that of GM@Fe_3_O_4_ and few aggregates can be observed, demonstrating the electrostatic attractions almost completely blocked. By contrast, the NMMG in 200 mm NaCl solution showed a size of ≈25 µm and the aggregates were obvious, indicating the more electrostatic interaction binding sites between Gelatin NPs and NM@Fe_3_O_4_.

### 2.3 In Situ Growth of Fe_3_O_4_ for Enhanced Mechanical Properties and Magnetothermal Effect

**Figure 4:**
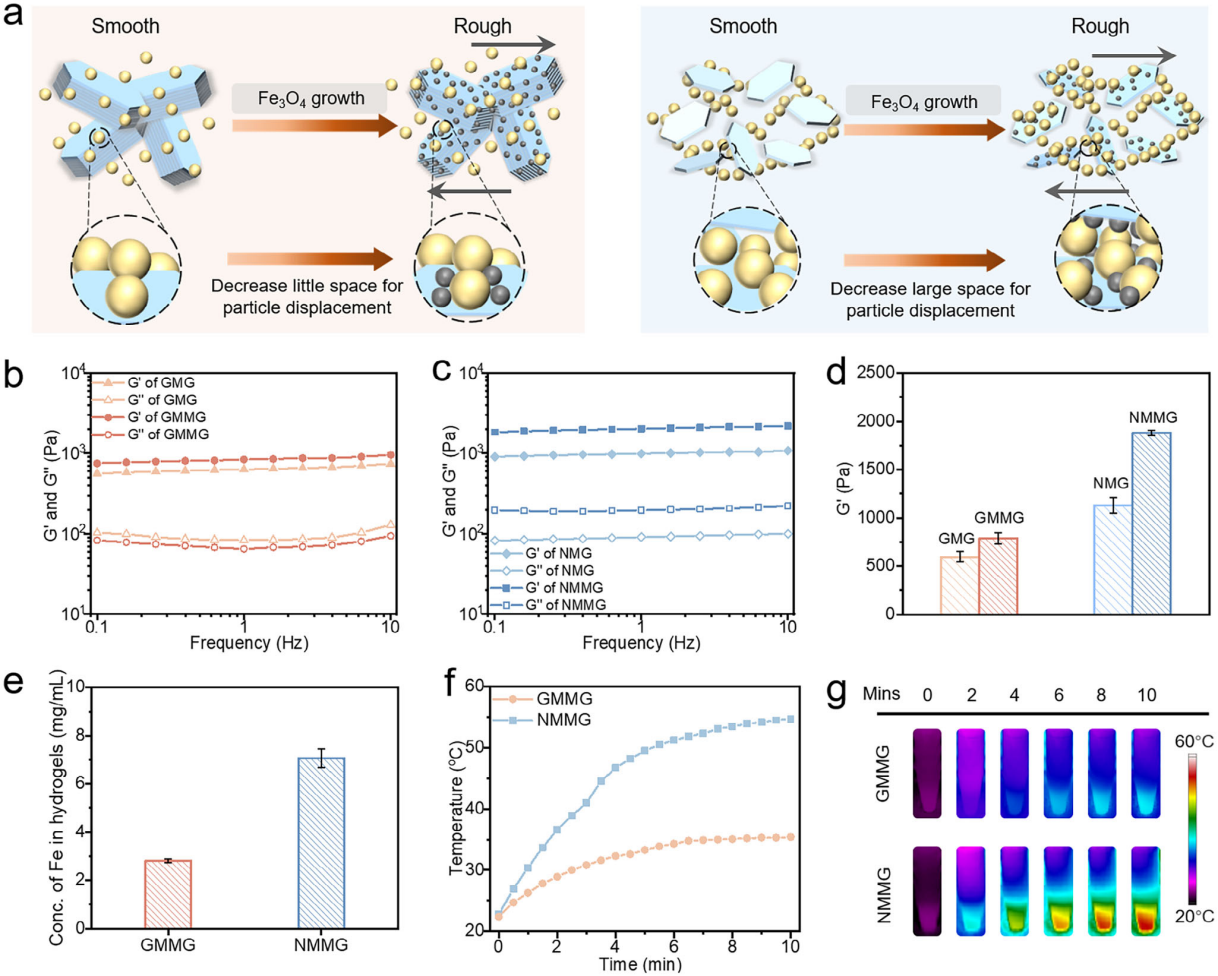
a) Schematic depiction of the enhanced gelation mechanism, where the Fe_3_O_4_ growth decreased the space of particles’ displacement. b,c) The rheological characterization and d) G′ values of CGs before and after in situ growth of Fe_3_O_4_. e) The concentration of Fe element in the GMMG and NMMG. f) Time‐dependent temperature change curves of CGs and g) corresponding thermal infrared images.

The surfaces of GM and NM were relatively smooth (Figure S7a,c, Supporting Information). By contrast, from the SEM images of Figure S7b,d (Supporting Information), the surface of GM@Fe_3_O_4_ and NM@Fe_3_O_4_ were relatively rough. In recent work, it has been reported that employing rough particles was beneficial to enhance mechanical properties in the construction of CGs.^[^ ^40^ ^]^ The schematic strengthening process of Fe_3_O_4_ reinforcing mechanical properties of CGs was shown in **Figure** **4**a, which indicated that Fe_3_O_4_ nanoparticles were favorable for the stability of GMMG and NMMG by restricting the particles’ migration and mobility of the colloidal chains. Owing to the roughness of Fe_3_O_4_, as depicted in Figure 4b–d, the G′ increased from ≈1100 Pa of NMG (NM colloidal gel without Fe_3_O_4_) to ≈1800 Pa of NMMG. While a weak enhancement from ≈600 Pa of GMG (GM colloidal gel without Fe_3_O_4_) to ≈800 Pa of GMMG in G′ was observed. Thus, we summarized that the Fe_3_O_4_ effectively hindered the migration between the building blocks and colloidal chains in the network during shear or vibration, thereby enhancing their mechanical performances. Notably, the difference in mechanical enhancement can be explained by the larger specific surface area of the NM@Fe_3_O_4_ (Figure S8, Supporting Information), which enabled nearly three times growth efficiency of Fe_3_O_4_ (Figure 4e) and better bonding capacity with Gelation NPs. Both of the GMMG and NMMG revealed significant superparamagnetism, and the saturation magnetization of GMMG was around the one third of NMMG, which was consistent to the results of iron oxide concentration (Figure S9, Supporting Information). The successful growth of Fe_3_O_4_ endowed them with magnetic heating properties. As demonstrated in Figure 4f,g, the NMMG rose above 50 °C within 10 min. By contrast, the temperature of GMMG rose still below 35 °C after 10 min. Meanwhile, Figure S10a–d (Supporting Information) revealed that the magnetic field intensity (20, 25 kA m^−1^) dependent heating temperature exhibited the same disparity in magnetothermal effect between the NMMG and GMMG. The stable magnetic heating change in several cycles (Figure S11, Supporting Information) revealed the feasibility of temperature control and multiple hyperthermia treatments of NMMG. Compared with GMMG, NMMG exhibited more promising magnetic heating performance by employing NM@Fe_3_O_4_ as building block, meeting the required temperatures of MHT with lower magnetic field intensities avoiding the potential side effects to surrounding normal tissues.

### 2.4 Mechanical Properties Characterization of NMMG and GMMG

**Figure 5:**
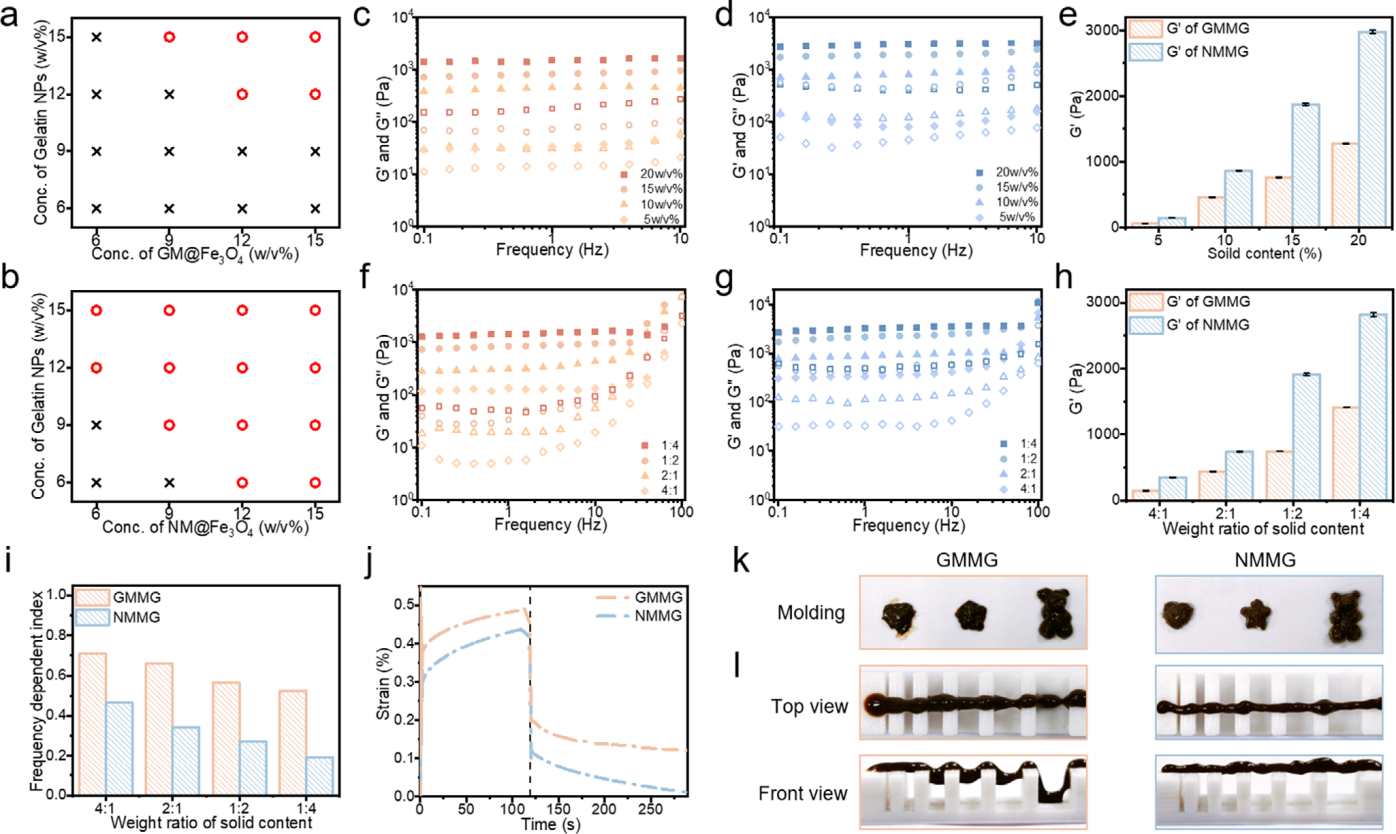
Rheological behavior comparison of binary composite CGs. Comparison of the gelation range of a) GMMG and b) NMMG (Black symbols: solution; Red points: hydrogel). Oscillatory frequency sweep tests for c) GMMG and d) NMMG under total solid mass varied from 5 to 20 w/v%. Representative Oscillatory frequency sweep tests of f) GMMG and g) NMMG with the different weight ratios varied from 4:1 to 1:4 at a total solid mass of 15 w/v%. The G′ of (e) the various solid content and h) different weight ratios of GMMG and NMMG. i) Frequency‐dependent index analysis of GMMG and NMMG. j) Creep tests of GMMG and NMMG under constant shear stress of 5 Pa followed by measuring residual strain upon stress removal for 3 min. Photographs showing k) mold shaping behavior and l) self‐supporting property of the GMMG and NMMG.

Furthermore, by controlling the solid concentrations, the bottom‐up assembly of CGs allowed for precise regulation of the mechanical properties as reported.^[^ ^61^ ^]^ By modulating the concentrations of Gelatin NPs and NM@Fe_3_O_4_ (GM@Fe_3_O_4_), the CG formation range was judged through the tube‐invest test and the inverted non‐flowing state indicates the gelation.^[^ ^62^ ^]^ **Figure** **5**a,b demonstrates that NMMG possessed a wider formation range compared to GMMG owing to its increased Gelatin NPs binding capacity (Figure S12, Supporting Information), which ensured the CGs stability and characteristics under broader circumstances thereby extending its application. Varying contents of GMMG and NMMG (5‐20 w/v%) were then investigated by frequency sweep rheological measurements. Notably, the G′ of NMMG surpassed that of GMMG at the same solid concentrations (Figure 5c,d), and the G′ of NMMG was ≈ 2900 Pa which was nearly twice times than that of GMMG at 20 w/v% (Figure 5e). The remarkable increase confirmed the enhancement of NM@Fe_3_O_4_ on the CGs’ characteristics. Subsequently, the CGs were fixed at the same concentration of 15 w/v% to explore the influence of the building blocks' weight ratio on CGs mechanical properties. For weight ratio ranging from 4:1 to 1:4 (mica composites: Gelatin NPs) in Figure 5f,g, frequency sweeps of these CGs revealed the G′ was increased as the increased addition of Gelatin NPs, which suggested increasing interactions between the building blocks. Nevertheless, in contrast to the rare change of G′ in GMMG when the weight ratio ranged from 2:1 to 1:2, the NMMG visually exhibited the rapidly increased and higher G′, which verified the increased contact area and binding capacity of the building blocks (Figure 5h). The frequency dependent indexes (FDI) of CGs were further quantified by calculating the slopes of the frequency‐sweep curves (Figure 5i). Upon increasing the weight ratios of Gelatin NPs, the FDI value of GMMG only decreased from ≈0.7 to ≈0.6. In contrast, the NMMG was more frequency‐independent, as evidenced by its FDI reduced from ≈0.5 to ≈0.2, implying NM@Fe_3_O_4_ can enhance the stiffness of the composite CGs. Considering other properties of the NMMG and GMMG, we chose a weight ratio of 1:2 at 15 w/v% for NMMG and GMMG as the optimal experimental ratio to perform the following experiments. The creep tests were performed to evaluate the ability of external force resistance. Upon release of the stress (5 Pa), GMMG displayed a slow recovery and the final strain maintained at 0.15% (Figure 5j). By contrast, NMMG displayed immediate recovery to the original stage, confirming the enhanced strength and viscoelasticity. Additionally, Figure 5k,l visually showed that NMMG possessed stronger self‐supporting ascribed to superior mechanical properties compared with GMMG. Owning to the reversibility of electrostatic interactions, GMMG and NMMG exhibited self‐healing behavior after undergoing destructive shearing. The NMMG revealed an immediate recovery of ≈90% while GMMG only recovered ≈77% after 90 s (Figure S13, Supporting Information).

### 2.5 Extrudability and Shape‐Fidelity of NMMG and GMMG

**Figure 6:**
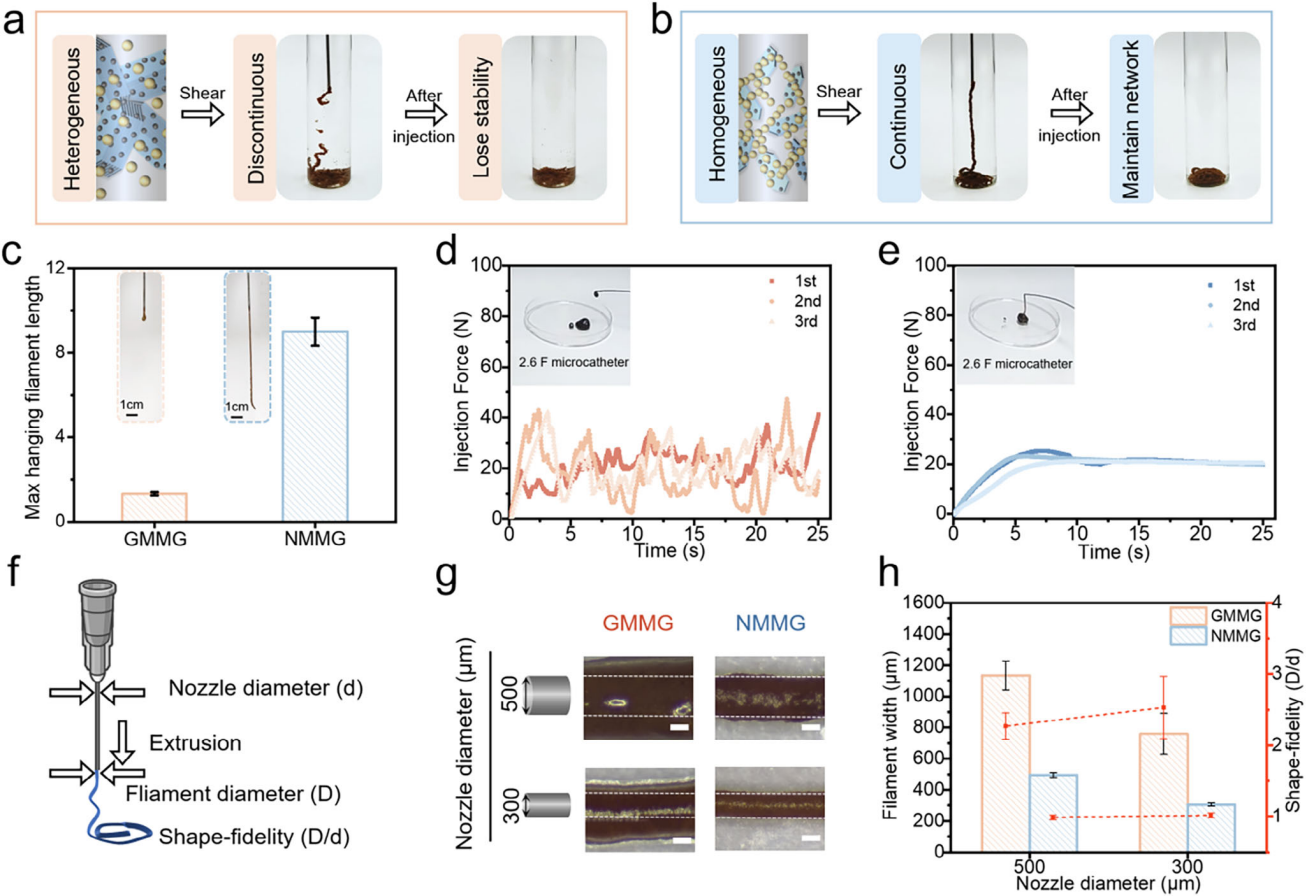
The extrudability and shape‐fidelity of the binary composite CGs (GMMG and NMMG). Macroscopic images extruded from an 18 G needle and schematic of the injection process through syringe of a) GMMG, b) NMMG. c) The filament extrusion tests of GMMG and NMMG. The injection force of d) GMMG and e) NMMG under 3 injection cycles through 2.6 F catheter. f) Schematic depiction of the shape‐fidelity assessed by extrusion expansion rate (D/d). g) photographs and h) quantification of the filament width during the extrusion. Scale bar: 200 µm.

CGs possessed shear‐thinning behavior, which was considered as the typical characteristic of injectability (Figure S14, Supporting Information). To determine extrudability of CGs for minimally invasive implantation procedures, the injectable properties were examined through clinical percutaneous needles. Both GMMG and NMMG were extrudable through an 18G needle, as depicted by photograph assessment (**Figure** **6**a,b). Meanwhile, the schematic illustrated the structural networks induced injection difference of GMMG and NMMG. In detail, the GMMG lost integrity being discontinuous, whereas NMMG could be injected from syringe to form a uniform hydrogel line and maintain the network after injection. Furthermore, filament extrusion tests were performed to investigate the injection performance (Figure 6c). Compared to the discontinuous droplets (≈1.3 cm) of GMMG, NMMG was extruded consecutively and smoothly, showing a larger average maximum length of the hanging filament (≈9.0 cm). We further investigated the injection force through clinical used 2.6 F catheter and 1 cc syringe combinations. The injection force curve of GMMG presented an obvious irregular fluctuation phenomenon (Figure 6d), showing instability and discontinuity due to the heterogeneous assembly. By contrast, a relatively stable injection force curve (Figure 6e) illustrated that the injection force (≈20 N) of NMMG was within a range that can be injected manually without the need for additional equipment (>50 N). The relationship between the injection performance and the CG's structural network was evaluated by assessing deformation parameters of extrusion expansion rate in the shape‐fidelity of the CGs during injection, where the value of D/d closer to 1 indicated better fidelity (Figure 6f). As a result, GMMG showed rather nonuniform filaments with the value of D/d significantly greater than 1 (Figure 6g,h), which was unable to retain its shape during injection. Nevertheless, NMMG presented continuous and homogeneous filaments even through the smaller needle (diameter of 300 µm) with the D/d closer to 1. From these results, the reversible network of NMMG allowed more compression during extrusion, thereby enabling more rapid network recovery after being squeezed out of the nozzle. In addition, we conducted injection studies using devices with varying internal diameters (16 G, 20 G, and 24 G) at a constant injection rate of 2 mL min^−1^. As shown in Figure S15 (Supporting Information), the injection forces for NMMG were consistently below 3 N, significantly lower than the manual injection force threshold (≈50 N),^[^ ^23^ ^]^ demonstrating its suitability for transcatheter and percutaneous injection procedures. In summary, due to the low injection force and appropriate shape‐fidelity, NMMG not only could be injected by hand via catheter but also owned stable mechanical properties, allowing the prospective application as an embolic agent.

### 2.6 In Vitro Occlusion Assessment of CGs and Lipiodol

**Figure 7:**
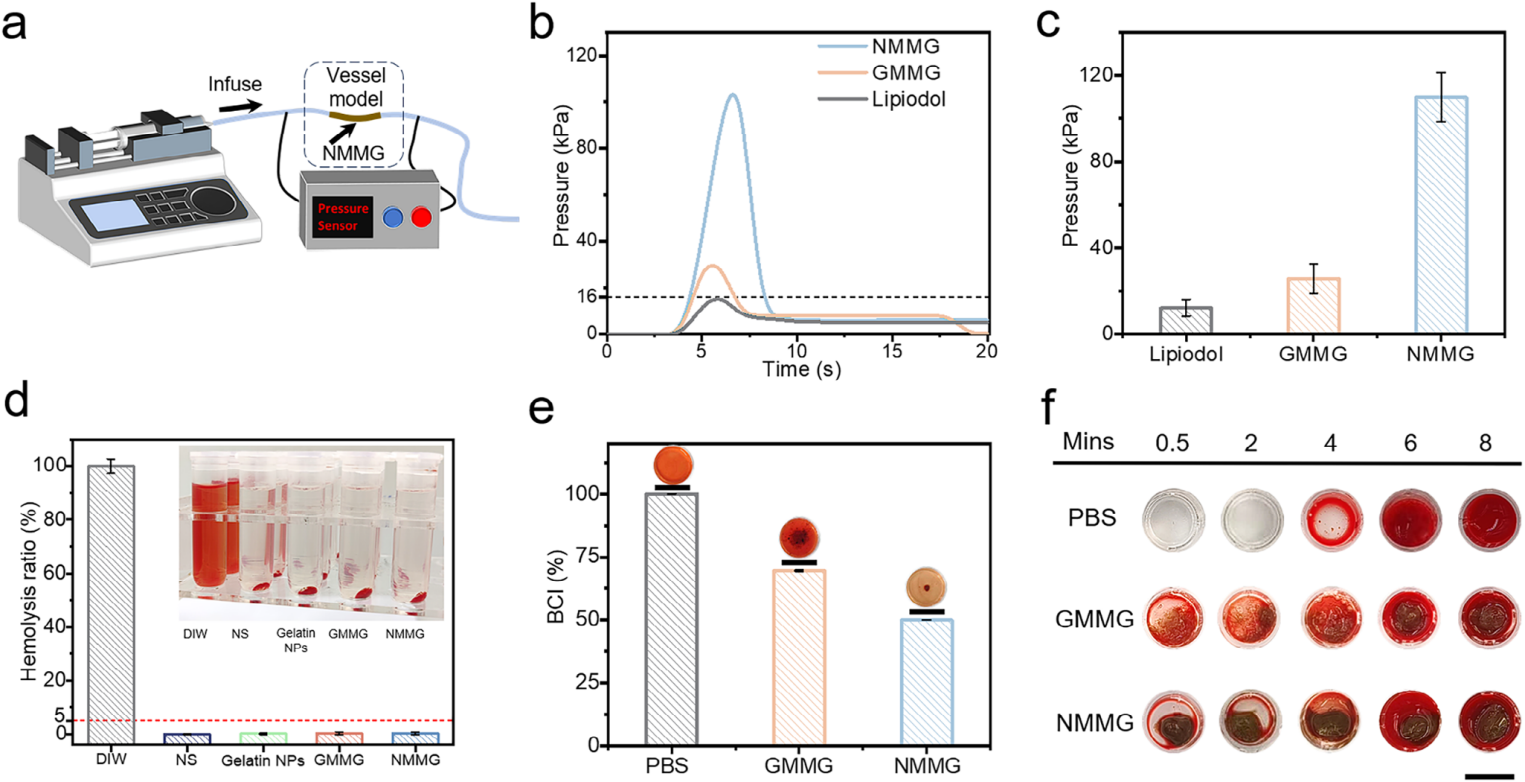
The evaluation of in vitro occlusion assessment, blood compatibility and thrombogenicity. a) Schematic for the displacement pressure measuring system. b) Representative pressure displacement curves of CGs and commercial Lipiodol, and c) a summary of the pressure required to displace. d) Hemolysis test of under diverse treatments and the corresponding photographs (inset). e) BCI (%) values of untreated group, GMMG and NMMG in vitro dynamic whole‐blood‐clotting. Scale bar: 80 mm. f) Time‐dependent blood clotting of GMMG and NMMG‐coated wells. Scale bar: 10 mm.

In addition to injectability, the GMMG, NMMG and clinically used Lipiodol for occluding a high‐pressure blood vessel was investigated by measuring the maximum pressure (**Figure** **7**a). As shown in Figure 7b,c, the ability of NMMG to withstand pressure (≈110 kPa) was much higher than physiological pressure (120 mmHg, ≈16 kPa)^[^ ^9^ ^]^ in vitro vascular occlusion model, which was approximately 4 and 9 times higher than GMMG and Lipiodol respectively, demonstrating the optimal ability of NMMG to withstand physiologic pressure. There was no sign of hemolysis with a hemolysis rate lower than 1% in NMMG and GMMG, which a hemolysis rate below 5% is considered permissible (Figure 7d). Whereafter, the blood biochemical analysis and cell viability of NIH‐3T3 incubated with NMMG experiment both demonstrated the excellent biocompatibility of NMMG (Figures S16,S17, Supporting Information). Then, thrombogenicity was investigated by evaluating the blood‐clotting index (BCI) from in vitro dynamic whole‐blood‐clotting test. As shown in Figure 7e, the BCI of GMMG was ≈75% with the swelling of the network. By contrast, BCI of NMMG was low as ≈50% and the blood was almost transparent. Moreover, the thrombogenicity test in Figure 7f showed that blood in contact with NMMG and GMMG fully coagulated at 6 min, the network of GMMG underwent swelling resulting in visible clusters in the substrate due to the weak mechanical properties, whereas the NMMG remained the stability of network. As indicated above, the exceptional mechanical performance and injectability suggested that NMMG was promising as an injectable embolic agent.

### 2.7 In Vivo MHT Treatment of NMMG

**Figure 8:**
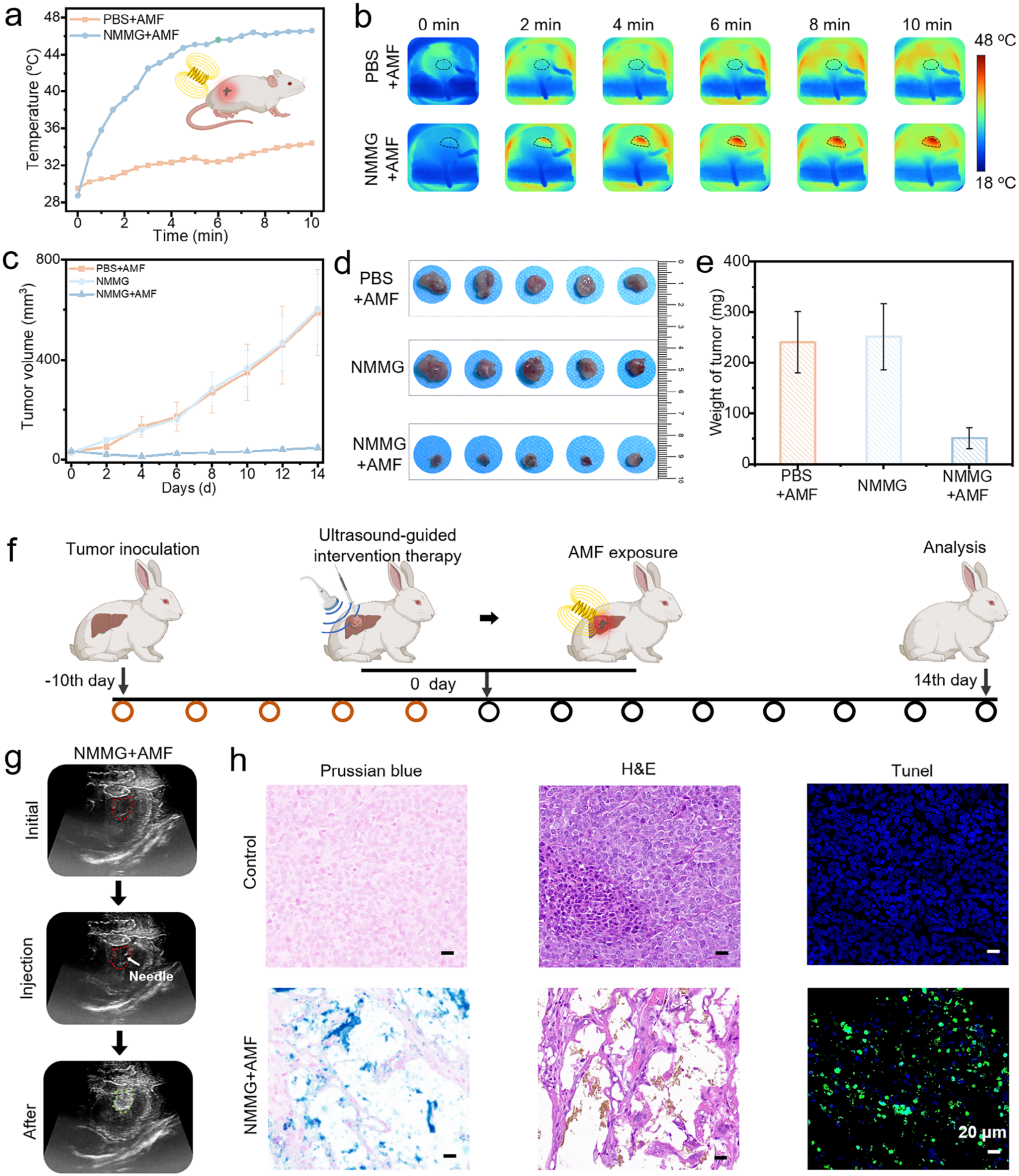
The evaluation of in vivo MHT treatment of NMMG. a) Temperature changes of tumor region from different groups under AMF and b) corresponding thermal infrared images. c) Tumor growth curves of mice during the various treatments. d) Photographs of tumors dissected from mice and e) tumor weight with different treatments on the 14th day. f) Schematic treatment process on VX2 tumor‐bearing rabbits. g) Ultrasound images of NMMG delivery process. The white arrow, red dash lines and green dash lines indicate needle, tumor, and NMMG, respectively. h) Prussian blue, H&E and Tunel staining of tumor slices collected from various groups. Scale bar: 20 µm.

A subcutaneous H22 tumor‐bearing mouse model was established to monitor and quantify the temperature elevation of hydrogels following intratumoral injection.^[^ ^63^ ^]^ The H22 tumor‐bearing mice were randomized into three groups (n = 5): (1) PBS+AMF group, (2) NMMG group and (3) NMMG+AMF group. When the mice were exposed to AMF, the NMMG+AMF groups displayed a sufficient magnetic hyperthermia effect with the temperature rose quickly to ≈42 °C within 4 min (**Figure** **8**a), and the temperature changes of the tumor site were recorded by the infrared thermal camera (Figure 8b). As shown in Figure S18 (Supporting Information), no significant body weight loss was observed between treated groups, indicating the safety of our treatments. Figures 8c–e and S19 (Supporting Information) show the rapid tumor growth rates in the PBS+AMF group and NMMG group. In contrast, the group of NMMG+AMF displayed effective inhibition of tumor growth.

Furthermore, VX2 tumor‐bearing rabbits were constructed to investigate the MHT effect under the ultrasound‐guided percutaneous puncture (Figure 8f). As shown in Figure 8g, under the ultrasound guidance, the NMMG was precisely injected into the tumor region of VX2 tumor‐bearing rabbits via a percutaneous needle. Then the rabbits were further exposed to the AMF. To visually evaluate the therapeutic efficacy, the rabbits were sacrificed and the tumor tissues were harvested for pathologic analysis. As shown in Figure 8h, Prussian Blue staining suggested that the NMMG was successfully injected into the tumor by the clinical percutaneous puncture under the ultrasound guidance. H&E staining image showed obvious necrosis and nuclear contraction in the NMMG+AMF group. Moreover, TUNEL staining analyses showed that NMMG‐mediated MHT could effectively induce tumor cell apoptosis (green fluorescence). These results demonstrated that the NMMG mediated MHT had an efficient HCC therapeutic effect via ultrasound‐guided minimally invasive interventional therapy.

### 2.8 Synergistic Therapy with TAE and MHT Treatment of NMMG

**Figure 9:**
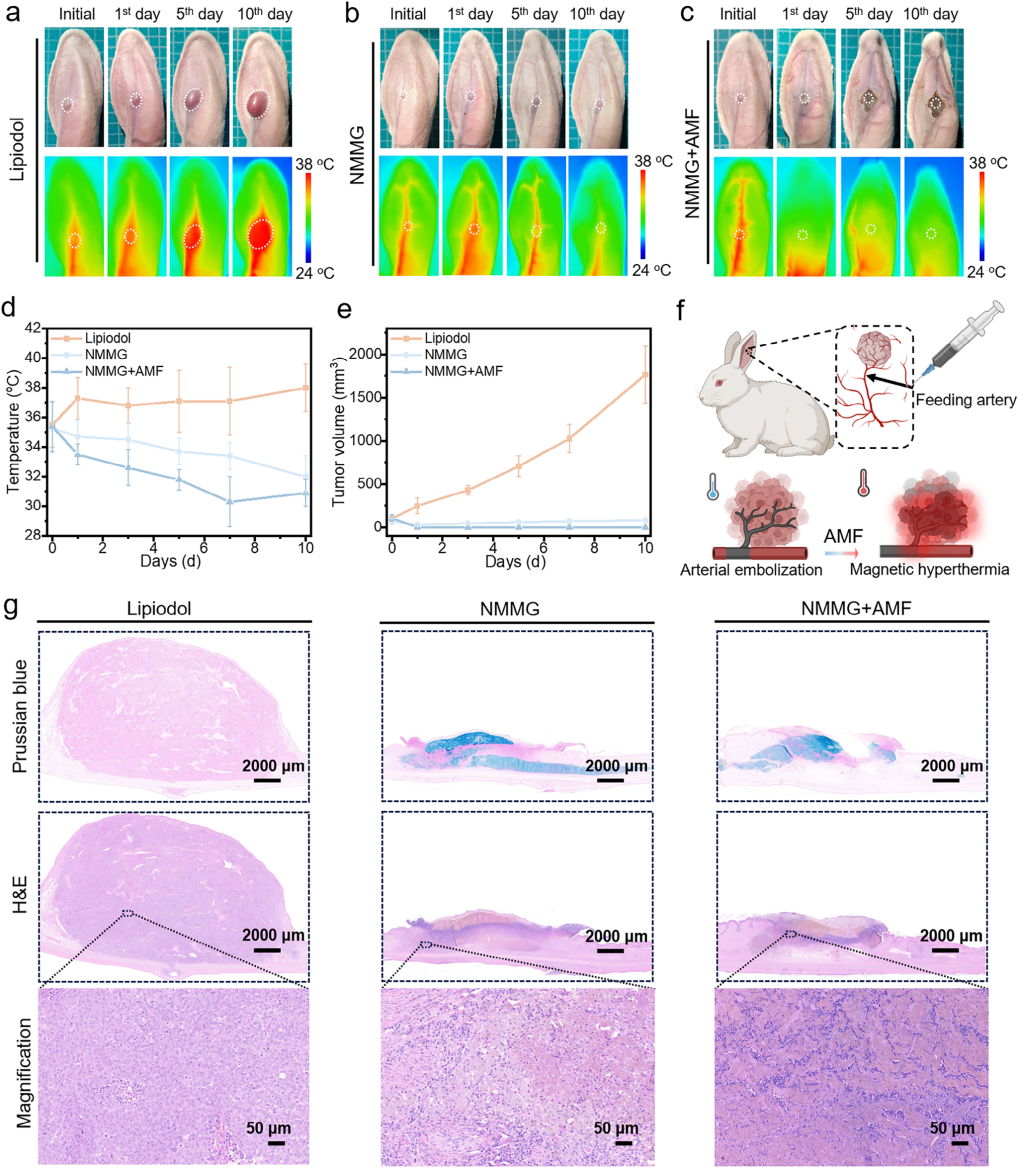
Evaluation of NMMG enhanced embolization in rabbit ears model of tumors. The photographs and corresponding infrared thermal images of rabbit ears before and after tumor treatments: a) Lipiodol group, b) NMMG group, c) NMMG+AMF group. The white circles indicated the embolized tumor. d) Temperature changes of rabbit ear tumor area with various treatments during the therapeutic process. e) Changes of rabbit ear tumor volume before and post‐embolization. f) Schematic of NMMG embolization combining magnetic hyperthermia enhanced the therapeutic effect. g) Representative Prussian Blue and H&E staining images of residual tumor from various groups.

A VX2 tumor‐bearing rabbit ear model was established to assess the synergistic therapeutic efficacy of NMMG‐mediated TAE combined with magnetic hyperthermia. The rabbits were randomly divided into three groups (n = 3): (1) Lipiodol group, (2) NMMG group, (3) NMMG+AMF group, then these agents were injected into the central auricular arteries of rabbits respectively. The temperature of tumor site in NMMG+AMF groups rose quickly to ≈44 °C, ensuring a consistent magnetically heated therapeutic (Figure S20, Supporting Information). As revealed by photographs and infrared thermal images of rabbit ears (**Figure** **9**a–c), the tumor exhibited obvious growth inhibition among NMMG and NMMG+AMF groups. In the NMMG group, slight punctate necrosis could be observed at the embolization site after 5 days with a temperature decrease in tumor region from ≈35 to ≈32 °C. The photographs and corresponding infrared thermal images (Figure 9d) of NMMG+AMF group exhibited that the blood flow disappeared in the distal end of the artery on 1st day, leading to a drop of temperature (≈30 °C as minimum temperature). After 10 days, the ear tissue became blackened and scabbed with significant shrinkage in tumor site, indicating that the combined treatment using embolization and MHT would induce a more severe locoregional necrotic effect compared to single embolization treatment. As shown in Figure 9e, the changes in tumor volume further demonstrated the optimal inhibitory effect of the NMMG+AMF group with almost complete elimination of tumor. Attributed to the injectability and the mechanical stability, the NMMG was able to enter into the targeted terminal of the vessels to realize endovascular embolization, which held great significance in the successfully precise heating of local tumor tissues with the occurrence of noticeable necrosis (Figure 9f).

According to the H&E images of the ears section (Figure 9g), the Lipiodol group presented a large number of active cancer cells. In the embolized tumor tissues, NMMG could be seen in the pathological section with Prussian Blue staining, proving the NMMG embolized the blood supply vessels of the tumor and led to regression of tumor. By analyzing the magnification of H&E staining, the NMMG+AMF group showed evident nuclear condensation or dissolution, whereas the NMMG group showed a part of the tumor cells survived with the risk of recurrence. These results indicated the TAE combined MHT with enhanced therapeutic effect held significant potential in the improvement of the tumor embolization. The in vivo metabolism of NMMG was evaluated using a rabbit ear embolization model. After post‐embolization, it exhibited that the blood flow disappeared in the central auricular arteries on 1^st^ day, leading to an obvious temperature loss in the entire rabbit ear. Histological analysis of arterial cross‐sections, particularly Prussian blue staining at the embolization site, confirmed the persistent presence of NMMG within the artery (Figure S21, Supporting Information). Additionally, NMMG (0.1 mL) was subcutaneously injected into the dorsal region of Balb/c mice to evaluate its in vivo degradation. After 10 days, the residual hydrogel exhibited no significant weight loss compared to the initial pre‐injection mass, indicating that the degradation of NMMG is a relatively slow process (Figure S22, Supporting Information).

## 3 Conclusion

In conclusion, we presented an interfacial reinforcement strategy for the design of CGs assembled by negatively charged NM@Fe_3_O_4_ and charge reversal Gelatin NPs, which can be injected through clinical catheter and mechanical stable in vessel to fulfill the needs of minimally invasive implantation. Due to the enhanced specific surface area and roughness, the NMMG possessed the continuous network structure and the more crosslinking sites to increase the stability of network thus showing the remarkable storage modulus more than ≈2 times than that of GMMG, and suitable injection force which applied the manual injection through 2.6 F catheter. Moreover, the NMMG presented excellent MHT performance with higher Fe_3_O_4_ content than GMMG. Further, in vivo tests demonstrated that NMMG exhibited considerable therapeutic efficacy for local lesions of mice and VX2 tumor‐bearing rabbits. Experimental results showed embolization of NMMG could induce obvious tumor growth inhibition. The combined treatment of vascular embolization and MHT would strongly enhance the treatment effect. Our study characterized the micro and nano‐scale building blocks in detail, bridging their structural characteristics with CGs properties. By regulating physico‐chemical properties of building blocks, this strategy will inspire the design of more CGs with robust mechanics and tailor‐made functionality, and further broaden the potential applications of CGs.

## 4 Experimental Section

### Materials

Gelatin A (from porcine skin, 300 Bloom), Iron (III) acetylacetonate (≥99.9%) and Poly(sodium 4‐styrenesulfonate) (PSS, Mw ≈ 70000) were purchased from Sigma‐Aldrich Co. Ltd. D‐(+)‐gluconic acid δ‐lactone (GDL, ≥99.0%), Glutaraldehyde solution (25%), Tetraethylene glycol (99%) and Glycine (>99%) were obtained from Aladdin Reagent Co. Ltd. Acetone (>99%) was purchased from Sinopharm Chemical Reagent Co. Ltd. Mica was obtained from Chuzhou Gerui Technology Co. Ltd. All chemicals were used without any purification unless otherwise specified.

### The Synthesis of GM@Fe_3_O_4_ and NM@Fe_3_O_4_

The exfoliation method of ground mica (GM) to nanosheet mica (NM) was according to the previous report,^[^ ^50^ ^]^ briefly, the GM was activated and intercalated by CTAB, and then the NM was obtained by ultrasonication. The in situ growth of Fe_3_O_4_ on the GM and NM was achieved by the thermal decomposition method. Typically, 200 mg of GM or NM and 200 mg of iron acetylacetonate were added to 50 mL of triethylene glycol. The mixture was heated to 300 °C at a rate of 3 °C min^−1^ and kept at 300 °C for 30 min. The obtained solution was centrifuged at 8000 rpm for 10 min and washed three times, then the GM@Fe_3_O_4_ or NM@Fe_3_O_4_ was obtained.

### Preparation of NMMG and GMMG

According to the previous report,^[^ ^29^ ^]^ Gelatin NPs were prepared by a two‐step desolvation method. To fabricate these magnetic CGs, a certain amount of Gelatin NPs and GM@Fe_3_O_4_ or NM@Fe_3_O_4_ were mixed by vortex at alkaline condition (pH≈12), and 80 mM GDL powder was subsequently added to induce gelation. Afterwards, the GMMG or NMMG was obtained. The CGs with different solids content and mass ratio of building blocks were synthesized by the above method.

### The Fluorescence Modification of Building Blocks

The preparation of Gelatin nanoparticles labeled by FITC was carried out as described before.^[^ ^30^ ^]^ A mass ratio of 1:10 was used between FITC and Gelatin nanoparticles. The aqueous solution of FITC was added to a dispersion of Gelatin nanoparticles at room temperature for 12 h under constant stirring. Thereafter, the dispersion of FITC‐Gelatin was washed by DIW four times. The preparation of GM@Fe_3_O_4_ or NM@Fe_3_O_4_ labeled by Rhodamine was carried out as described. Rhodamine B and APTES were added into 5 ml of dimethylsulfoxide with continuous stirring for 1 h at room temperature. Then 6.5 mg of GM@Fe_3_O_4_ (or NM@Fe_3_O_4_) were added into the above solution with continuous stirring for 12 h. The mixtures were centrifuged at 8000 rpm for 10 min and washed until the supernatants were transparent.

### Characterization of Colloidal Assembly by Dynamic Light Scattering (DLS)

The changes of the suspensions assembled by building blocks as a function of time were measured by DLS (Malvern Zetasizer, MS2000), containing both types of nanoparticles at various mass ratio of magnetic mica composites to Gelatin NPs. All groups were tested in deionized water. Meanwhile, the influence of ionic strength on assembly of oppositely charged Gelatin NPs and magnetic mica composites was investigated by increasing the salt concentration (0, 100, and 200 mm NaCl). After the building blocks mixed in salt solutions, DLS was also used to monitor the change of particle size of mixture dispersion with different ionic strength.

### Characterizations

The morphologies were observed by scanning electron microscope (Zeiss Supra 40, Germany) and transmission electron microscope (Hitachi HT 7700). The particle size was evaluated by a dynamic light scattering instrument (Malvern Zetasizer, MS2000). XRD pattern was obtained using a Philips X’ Pert Pro SUPER instrument (Netherland). The structures of the CGs were visualized by inverted fluorescence microscope (Yuescope, YIB510FL). The rheological behavior was performed on the TA Discovery HR10 rheometer with a gap distance of 1000 µm. The injection force of CGs was measured using a mechanical testing system (Instron 5965, USA). The magnetic heating experiment was performed using high frequency heating equipment (Shuangping SPG, China) and recorded by infrared thermal imager (Fluke Ti10, USA).

### Rheological Characterizations of Colloidal Gels

The viscoelastic properties of NMMG and GMMG were characterized by frequency sweep measurement with frequency from 0.1‐100 Hz at a constant strain of 1%. The frequency‐dependent index (FDI) was defined as the slope of the curve of frequency sweep of colloidal gels. Self‐healing properties were evaluated quantitatively by monitoring the evolution of storage modulus (G′) and loss modulus (G′′) of colloidal gels during cycles of destructive shearing (oscillatory strain sweep with strain ranging from 0.1% to 500% at a constant frequency of 1 Hz for 100 s) and recovery (strain of 0.1% and frequency of 1 Hz for 100 s). To evaluate capacity of resistance to external force, creep experiments were performed under a constant shear stress of 5 Pa for 2 min followed by measuring residual strain upon stress removal for 3 min. The shear‐thinning behavior of colloidal gels was investigated by alternating shear rate of low (0.01 s^−1^) to high (100 s^−1^) at a fixed frequency (1 Hz) and strain (0.01%).

### Injection Performance Testing of Colloidal Gels

The equipment for testing the injection force was designed and carried out with 2.6 F medical catheter to test the injection force of NMMG and GMMG. The injection force of CGs at a predetermined flow rate was tracked in real‐time by the Bluehill version 3 software. The hanging filament test was performed with 18 G needle. For fidelity of the hydrogels, different nozzle diameters (300, 500 µm) were used to test the injectability. Briefly, the CGs were placed into 1 ml plastic syringes that were fixed vertically in a customized mold under compression mode, following the extrusion fell on the glass substrate. The width of extrusion (filament diameter) was measured and the extrusion expansion rate was defined with dividing filament diameter by nozzle diameter.

### In Vitro Occlusion‐Displacement Test

The ability of NMMG and GMMG in withstanding pressures relevant to physiology was assessed through an in vitro occlusion model. A tube containing 1 mL of colloidal hydrogel was utilized to simulate the embolization of a blood vessel, followed by a continuous infusion of PBS at a consistent rate of 70 mL min^−1^. Pressure changes during material displacement were meticulously monitored using a pressure sensor. The highest pressure of each material was recorded during the displacement. All the groups underwent three experimental tests and were summarized for analysis.

### Establishment of Tumor Bearing Mice/Rabbits and Magnetic Hyperthermia Therapy by NMMG

To establish tumor‐bearing mice, Balb/c female mice (6–8 weeks) received a subcutaneous injection of the H22 cells. Ten days after tumor cells inoculation, tumor‐bearing Balb/c female mice were divided into three groups (n = 5) for the evaluation of the MHT as follows: (1) PBS+AMF group; (2) NMMG group; (3) NMMG+AMF group. During the magnetic heating process, temperatures within the tumor region were monitored using infrared thermal imaging camera. Subsequently, both the mice's body weight and tumor size were recorded every other day. After 14 days, the mice were euthanized, the tumors were weighed and photographed.

To establish the VX2 tumor model rabbit, the VX2 tumor tissue suspensions were directly implanted into the livers of rabbits using 18‐gauge needle. On the 10^th^ day after implantation, all the VX2 tumor bearing rabbits were evaluated using computed tomography (CT). After the successful establishment of the VX2 tumor‐bearing rabbits, they were applied to conduct the ultrasound‐guided minimally invasive intervention therapy under the laparoscope guidance, dividing into 2 groups (n = 3) randomly including control group and NMMG+AMF group. Then the rabbits were anesthetized and injected with NMMG by ultrasound‐guided (Mindray Z70 Vet) percutaneous puncture. After 14 days, the rabbits were sacrificed and analyzed.

### In Vivo Degradation Tests of NMMG

To evaluate the metabolism of NMMG in feeding artery, the New Zealand white rabbit was anesthetized and then NMMG was injected into central auricular artery. The embolized rabbit ears were photographed on the 0, 1^st^ day after implantation respectively. The temperature of the ears was also recorded with an infrared thermal imager. On the 10^th^ day, rabbits were euthanized and the embolized ear were retrieved. The ears were fixed in formalin and embedded in paraffin, staining with H&E, Masson trichrome and Prussian blue respectively.

### In Vivo Combined Treatment of Tumor Bearing Rabbit Ears

To build a rabbit ear tumor model, the VX2 tumor tissue suspensions were injected near the central ear marginal arteries. After the tumors grew ≈ 100 mm^3^, the rabbits were anesthetized and NMMG was injected into the tumor's blood supply artery with a syringe. The same injection method of Lipiodol was used with the same volume. Subsequently, the rabbits of NMMG were exposed to AMF for combined treatment of embolization and MHT. The temperature changes and necrotic states of rabbit ears were monitored by infrared thermography and digital photographs during the treatment period. After 10 days, all the rabbits were sacrificed to evaluate the therapy efficacy, the tumor tissues were collected for H&E staining and Prussian blue staining analysis. All animal experiments were performed following the recommendations in the Guide for the Care and Use of Laboratory Animals of the National Institutes of Health, which were approved by the Institutional Animal Care and Use Committee of Hefei University of Technology (No. HFUT20211019001).

## Conflict of Interest

The authors declare no conflict of interest.

## Author Contributions

X.L. and S.C. contributed equally to this work. Y.S., L.D. and Y.L. conceived the idea and supervised the project. X.L. and S.C. planned and performed the experiments, collected and analyzed the data. B.C., X.Y., R.Q., J.H., B.Q.C., H.X., T.Z., L.D. assisted with the experiments and characterizations. X.L., S.C., X.Y., Y.S., L.D. and Y.L. co‐wrote the manuscript. All authors discussed the results and commented on the manuscript.

## Supporting information



Supporting Information

## Data Availability

The data that support the findings of this study are available from the corresponding author upon reasonable request.;
