# Supplementary material for: Interfacial Roughness‐Reinforced Magnetic Nanosheet Colloidal Gels for Stable Embolization and Magnetothermal Therapy of Hepatic Tumor
Source: Adv Sci (Weinh). 2025 Aug 13;12(36):e07096. doi: 10.1002/advs.202507096 (PMC12463041; doi:10.1002/advs.202507096)
Supplement: Supplementary file 1 — Supporting Information [file ADVS-12-e07096-s001.docx]

**Supporting information**

**Interfacial Roughness-Reinforced Magnetic Nanosheet Colloidal Gels for Stable Embolization and Magnetothermal Therapy of Hepatic Tumor**

*Xingyu Liu^1†^, Sheng Chen^1†^,* *Yonghong Song^1*^, Bing Chen^1^, Xu Yan^1^, Rui Qiu^1^, Jinlong Hu^3^, Baoqiang Cao^3^, Hanye Xing^1^, Tao Zhou^1^, Liang Dong^2*^, and* *Yang Lu^1*^.*

- ^1^Key Laboratory of Value-Added Catalytic Conversion and Reaction Engineering, School of Chemistry and Chemical Engineering, Hefei University of Technology, Hefei, Anhui, 230009, PR China.
- ^2^Hangzhou Institute of Medicine (HIM), Chinese Academy of Sciences, Zhejiang Cancer Hospital, Hangzhou, Zhejiang, 310018, PR China.
- ^3^Department of General Surgery, Department of Ultrasonics, Department of Interventional Radiology, Anhui No. 2 Provincial People’s Hospital, Hefei, Anhui, 230041, PR China.
- *^*^*Correspondence should be addressed to Yonghong Song, Liang Dong and Yang Lu.
- E-mail: songyh@hfut.edu.cn.; dongliang@him.cas.cn; yanglu@hfut.edu.cn.
- **This PDF file includes:**
- Figs. S1 to S22
- References: 1-4

**Supplementary Figures**


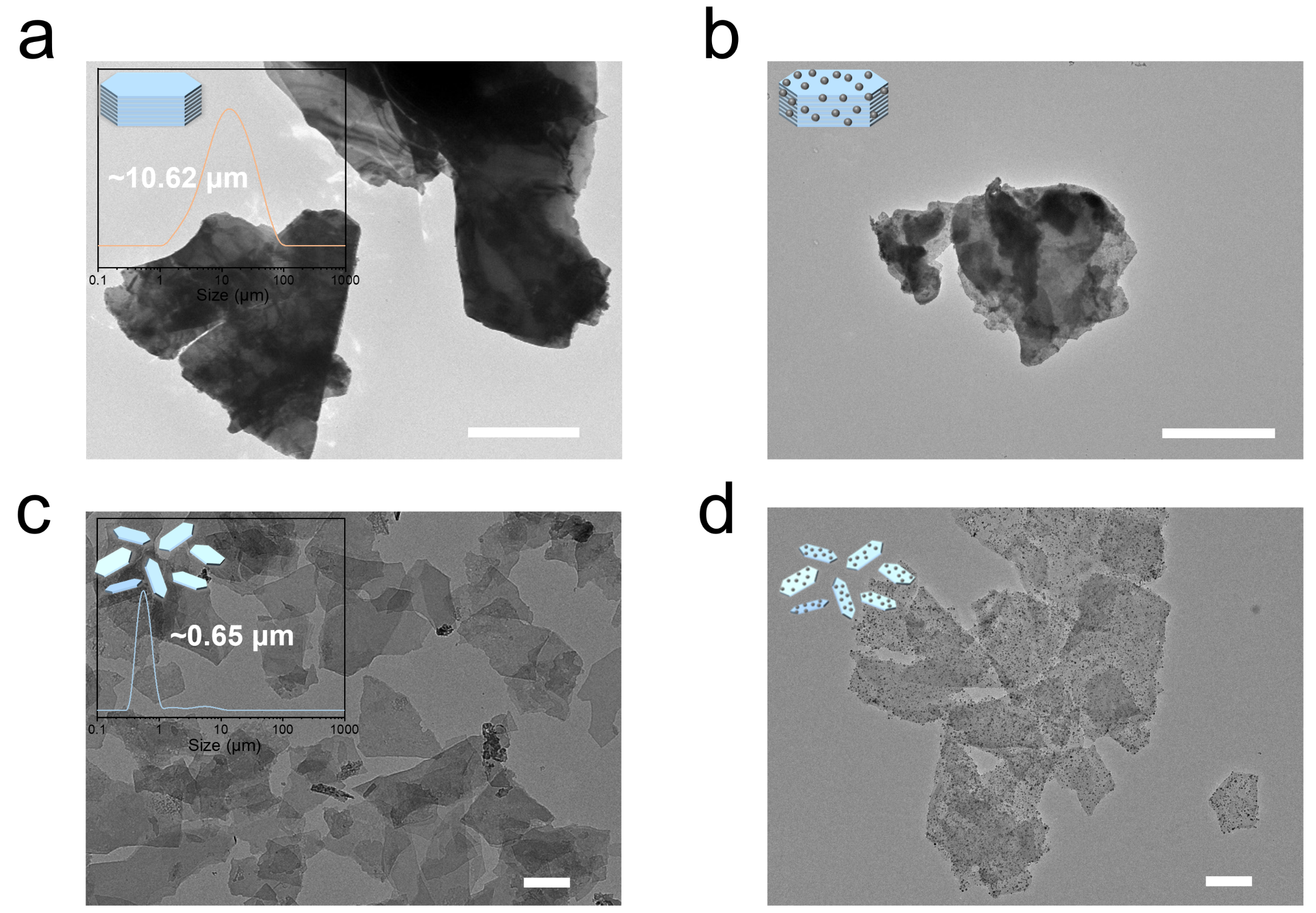


**Figure S1**. (a-b) Representative the TEM images of ground mica (GM) and magnetic ground mica (GM@Fe_3_O_4_). Scale bar: 5 μm. (c-d) Representative the TEM images of nanosheet mica (NM) and magnetic nanosheet mica (NM@Fe_3_O_4_). Scale bar: 500 nm.


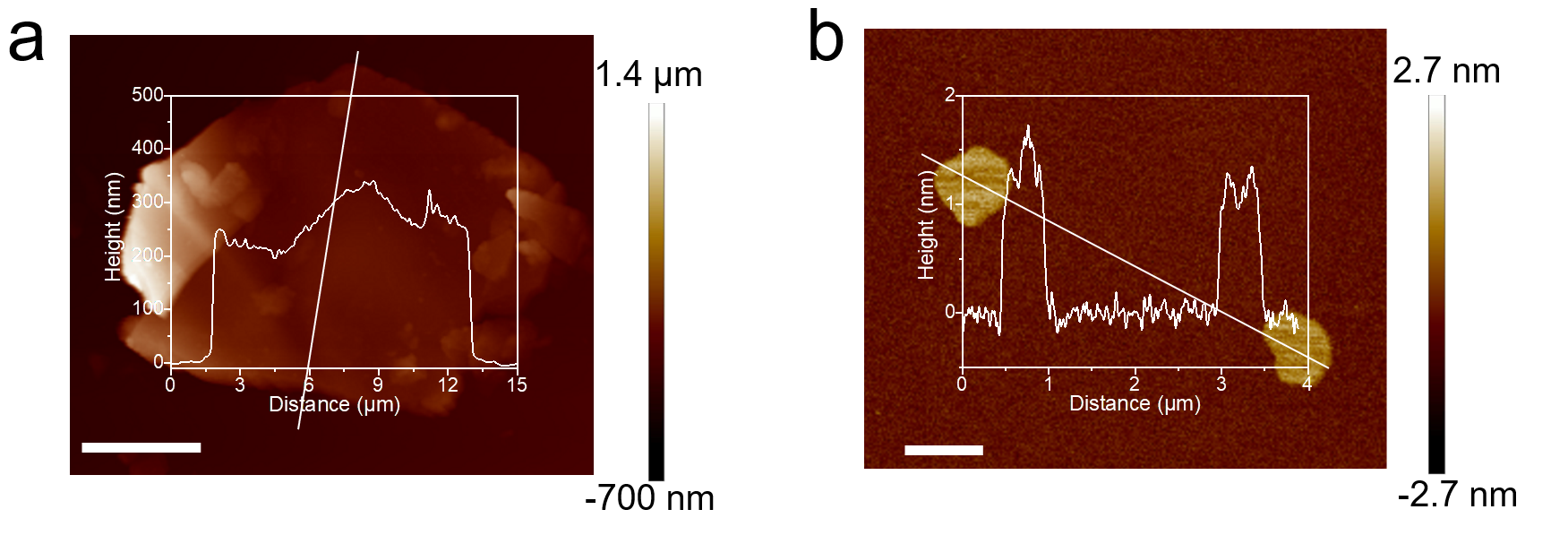


**Figure S2.** The AFM height images of (a) GM and (b) NM, respectively. Scale bar: 4 μm in (a) and 500 nm in (b). Insert displayed the thickness of (a) GM and (b) NM measured respectively by AFM.


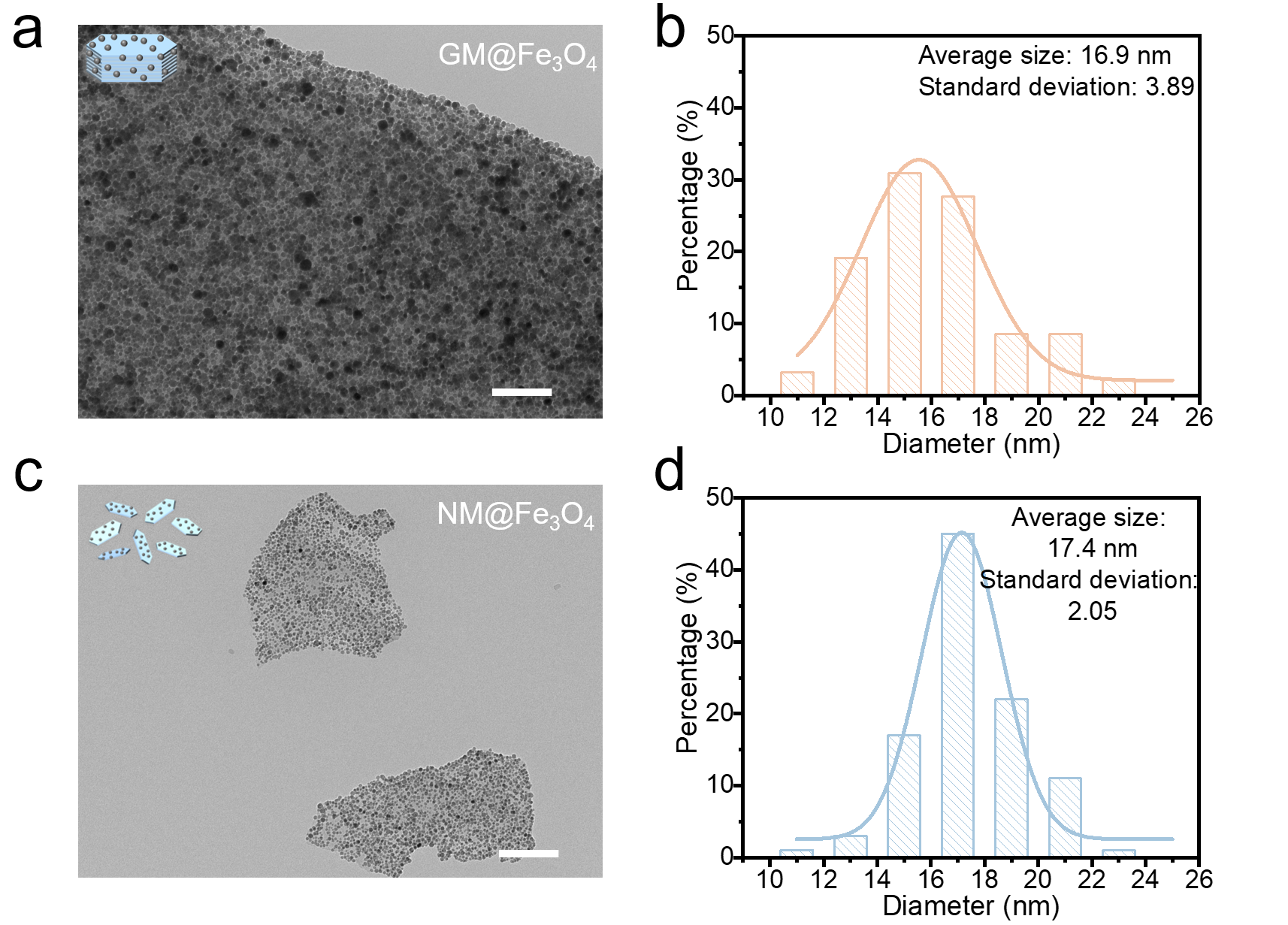


**Figure S3.** The TEM and corresponding size distributions of iron oxide nanoparticles on the (a-b) GM@Fe_3_O_4_ and (c-d) NM@Fe_3_O_4_. Scale bar: 200 nm.


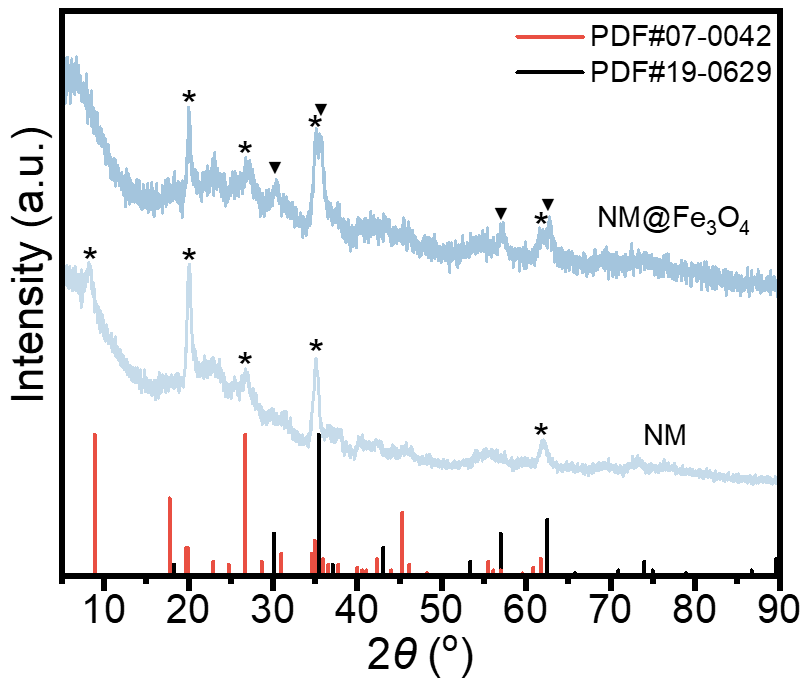


**Figure S4.** XRD pattern of NM and NM@Fe_3_O_4_. The asterisks symbol (*) presented the characteristic peaks of NM corresponding XRD standard card (PDF#07-0042).^[1, 2]^ The solid black inverted triangles symbol (^▼^) presented the characteristic peaks of Fe_3_O_4_ corresponding XRD standard card (PDF#19-0629).^[3, 4]^


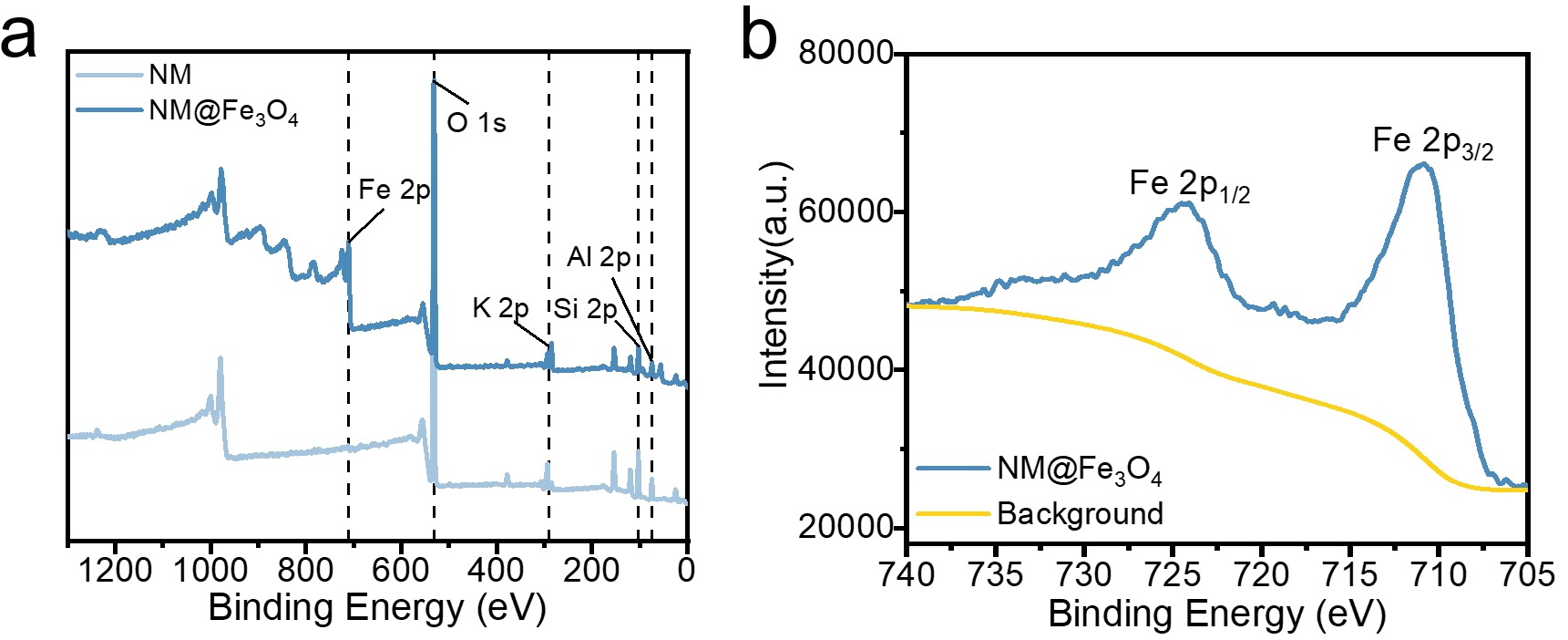


**Figure S5.** (a) XPS survey spectrum of NM and NM@Fe_3_O_4_. And (b) high-resolution of Fe 2p of NM@Fe_3_O_4_.^[3-4]^


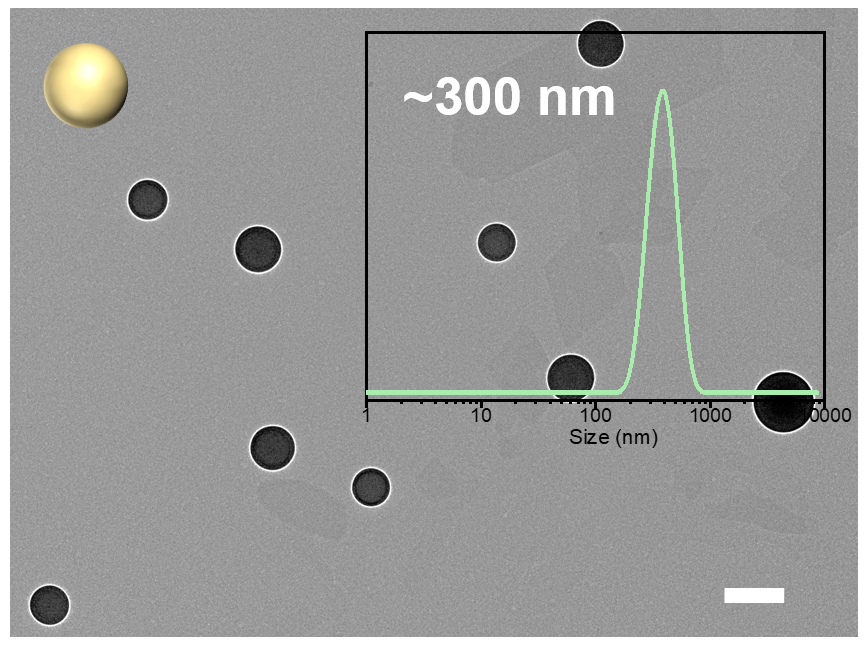


**Figure S6.** The TEM image and particle size distribution (insert in right) of Gelatin NPs. Scale bar: 500 nm.


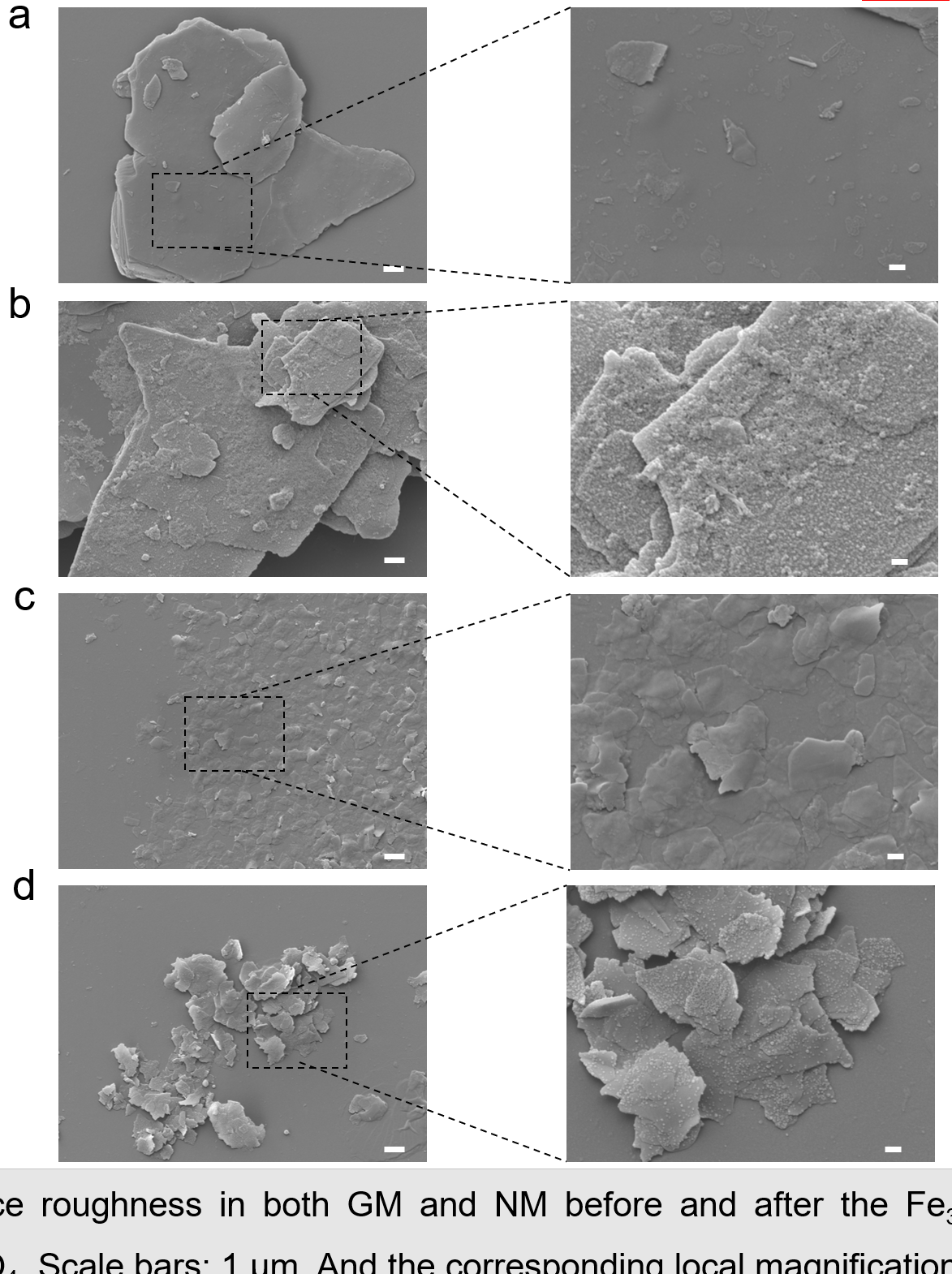


**Figure S7.** The changes of surface roughness in both GM and NM before and after the Fe_3_O_4_ modification. SEM images of (a) GM, (b) GM@Fe_3_O_4_, (c) NM, (d) NM@Fe_3_O_4_. Scale bars of left lines: 1 μm. Scale bars of right lines: 200 nm.


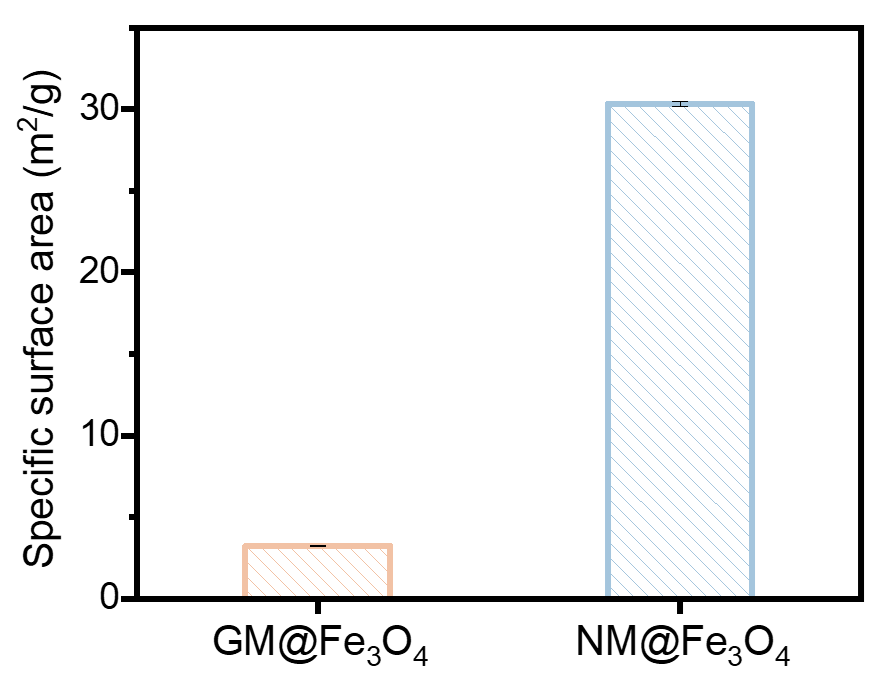


**Figure S8.** The corresponding specific surface area of GM@Fe_3_O_4_ and NM@Fe_3_O_4_. Specific surface area of those mica composites was determined by LASER granulometric method, which was widely used in characterization of powder materials.


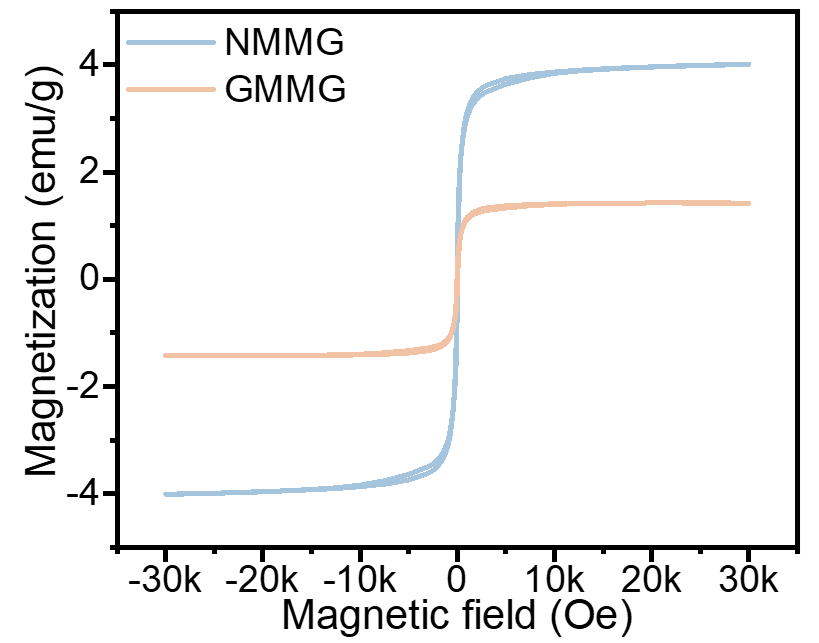


**Figure S9**. The magnetization curve of GMMG and NMMG.


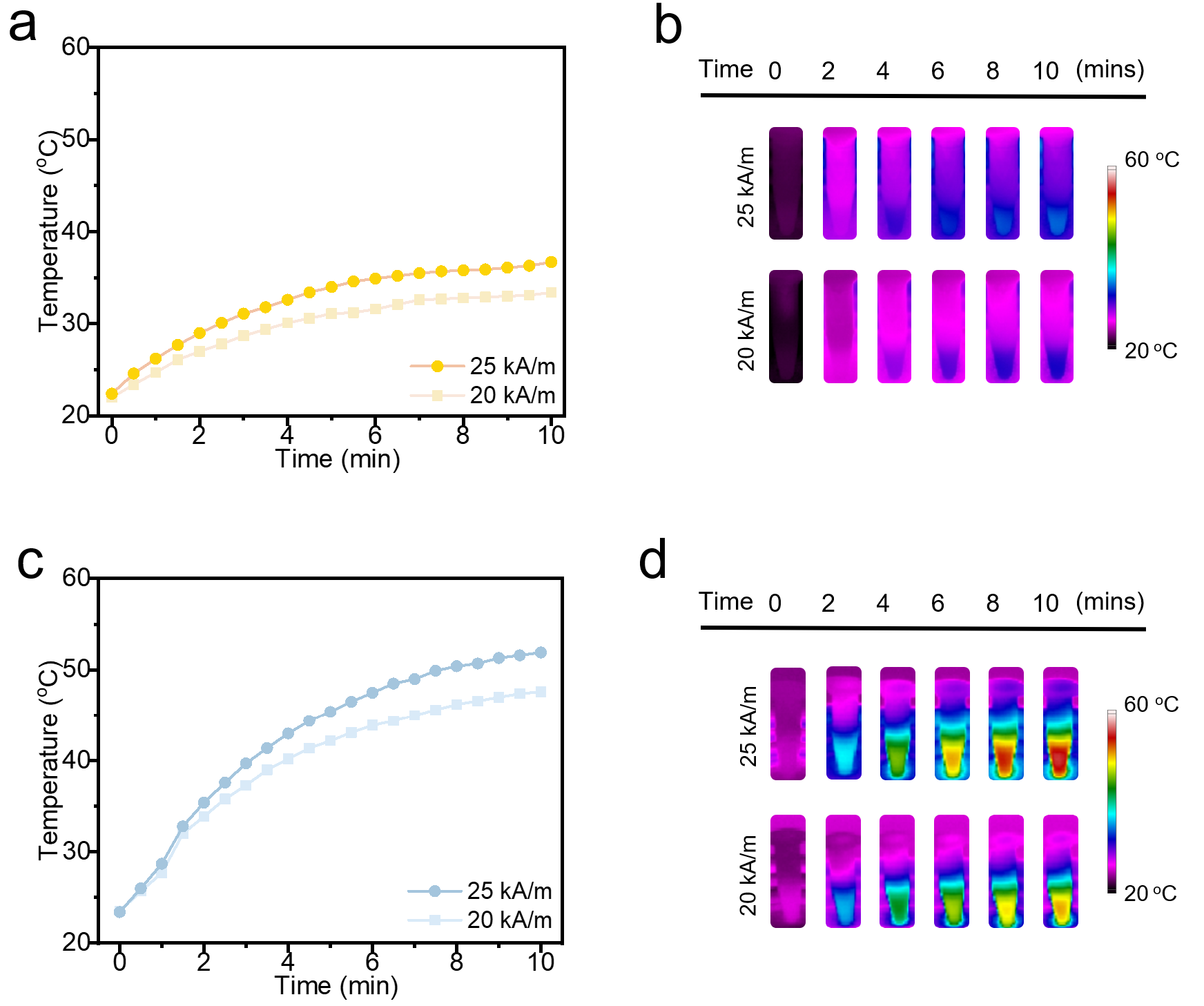


**Figure S10.** Magnetic field intensities related temperature change curves of (a) GMMG and (c) NMMG, (b) and (d) were representative thermal images. Both of NMMG and GMMG were synthesized with the ratio of negatively charged building blocks to positively charged building blocks equaled to 1:2.


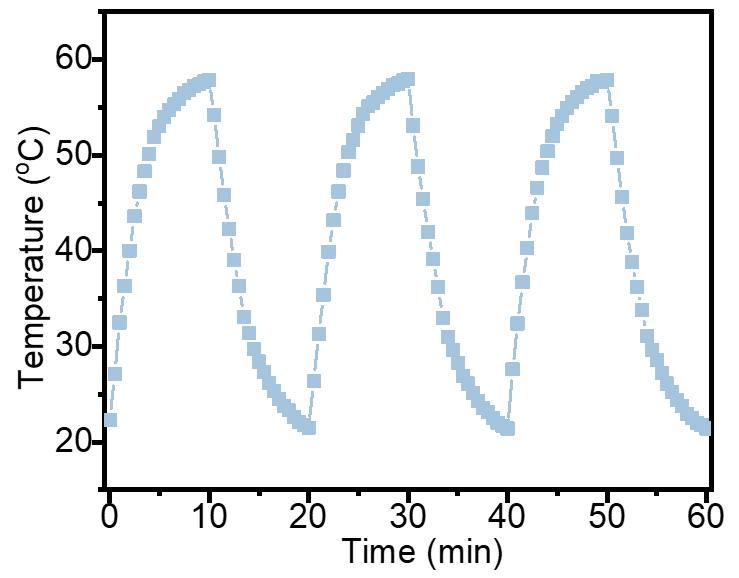


**Figure S11.** The temperature change of NMMG under the stimuli of AMF (H = 30 kA/m) for 10 min, then AMF was turned off for 10 min and this process was repeated for 3 times.


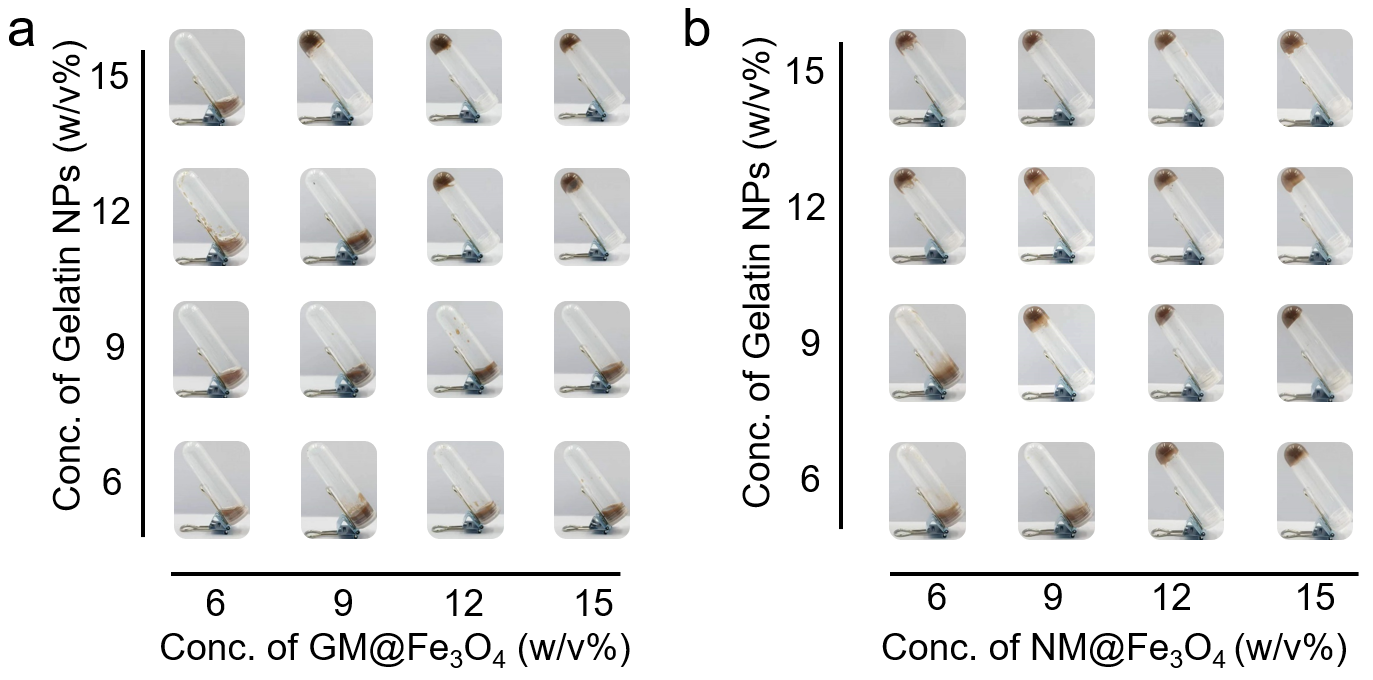


**Figure S12.** The physical state diagram for the mixture of opposite charged building blocks mediated sol-gel transition.


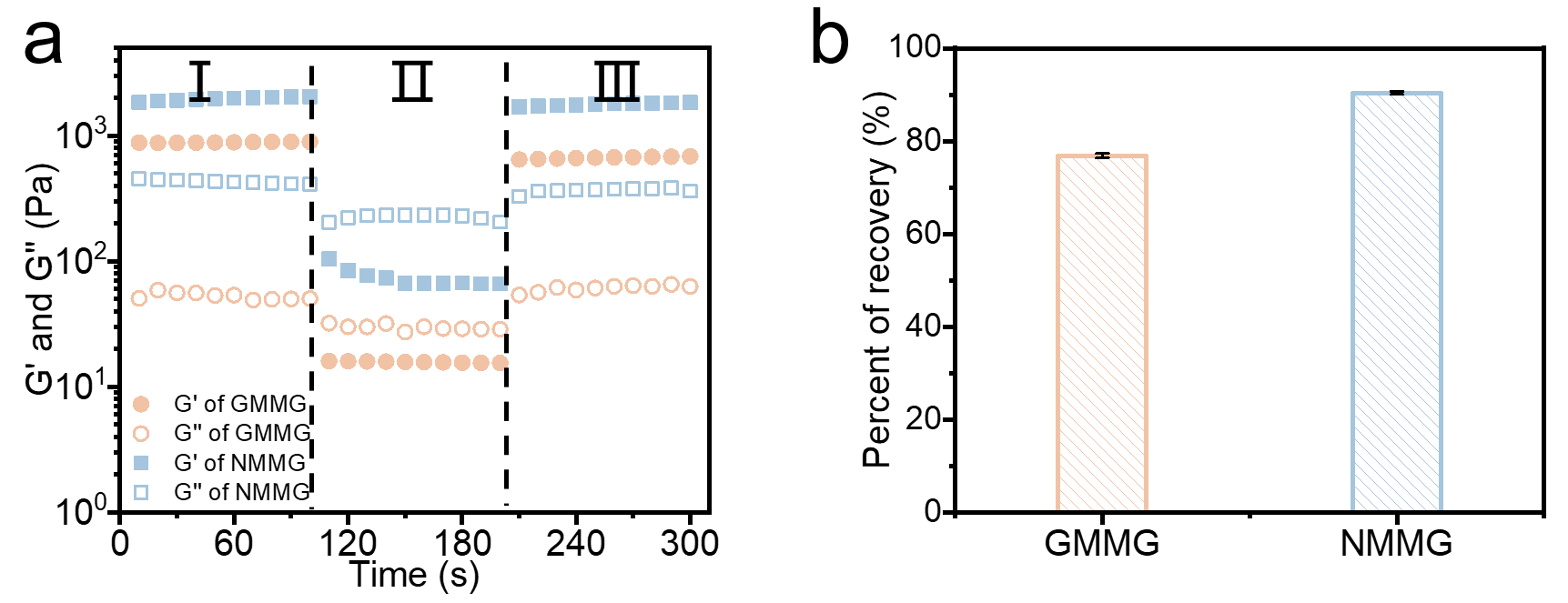


**Figure S13.** (a) Self-healing behaviors of GMMG and NMMG cycled between 1% and 500% strains. (b) The recovery percentage of storage modulus (G′) after binary composite colloidal gels network destruction.


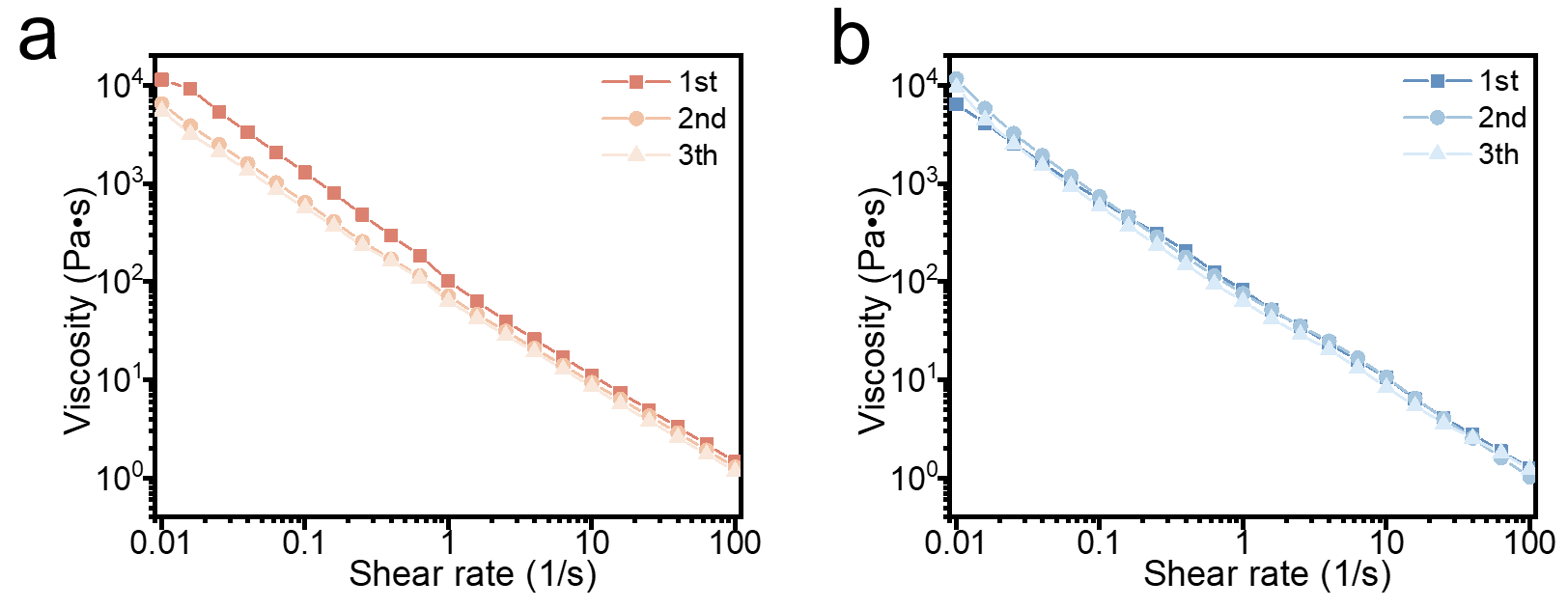


**Figure S14.** The shear-thinning behavior of colloidal gels: (a) GMMG and (b) NMMG.


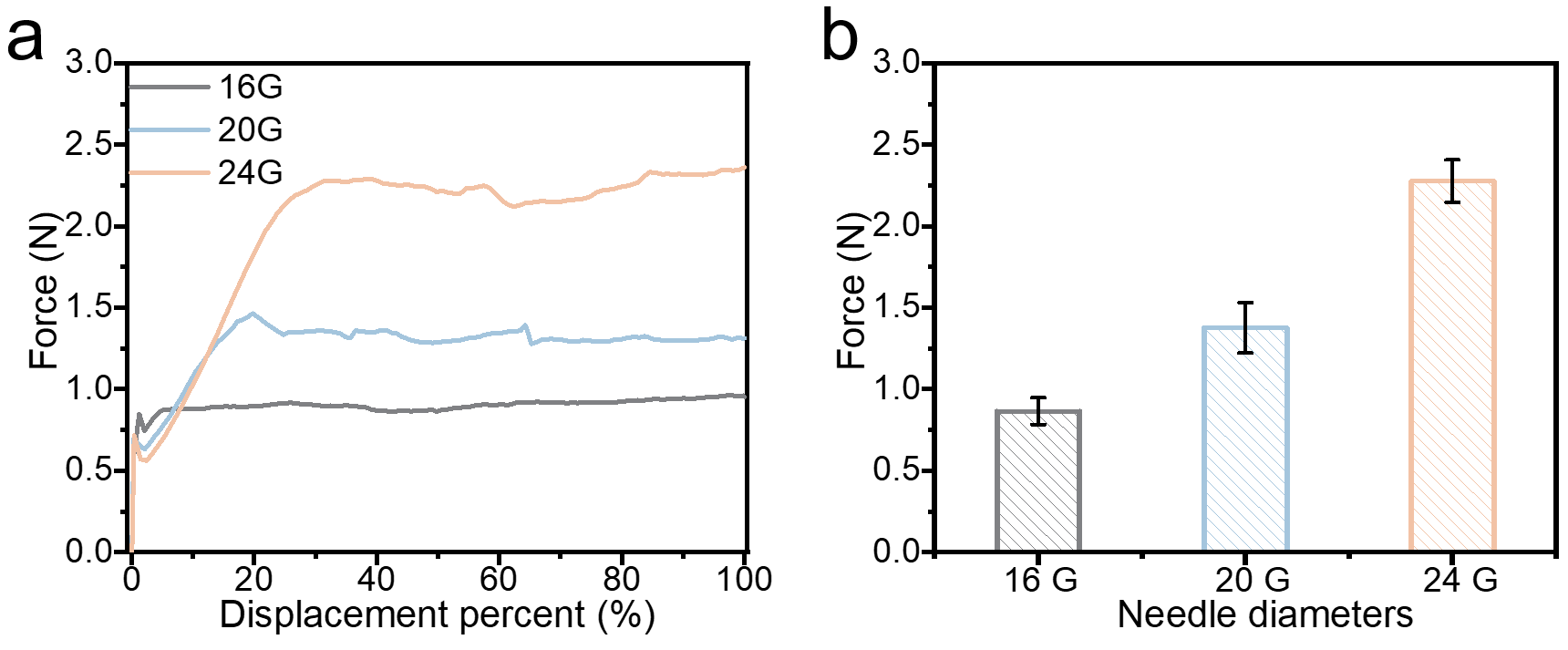


**Figure S15.** (a) Representative injection force curves of NMMG injected through percutaneous needles (16 G, 20 G, and 24 G) at a constant injection rate of 2 mL/min. (b) Quantitative comparison of injection forces for NMMG using percutaneous needles of varying internal diameters.


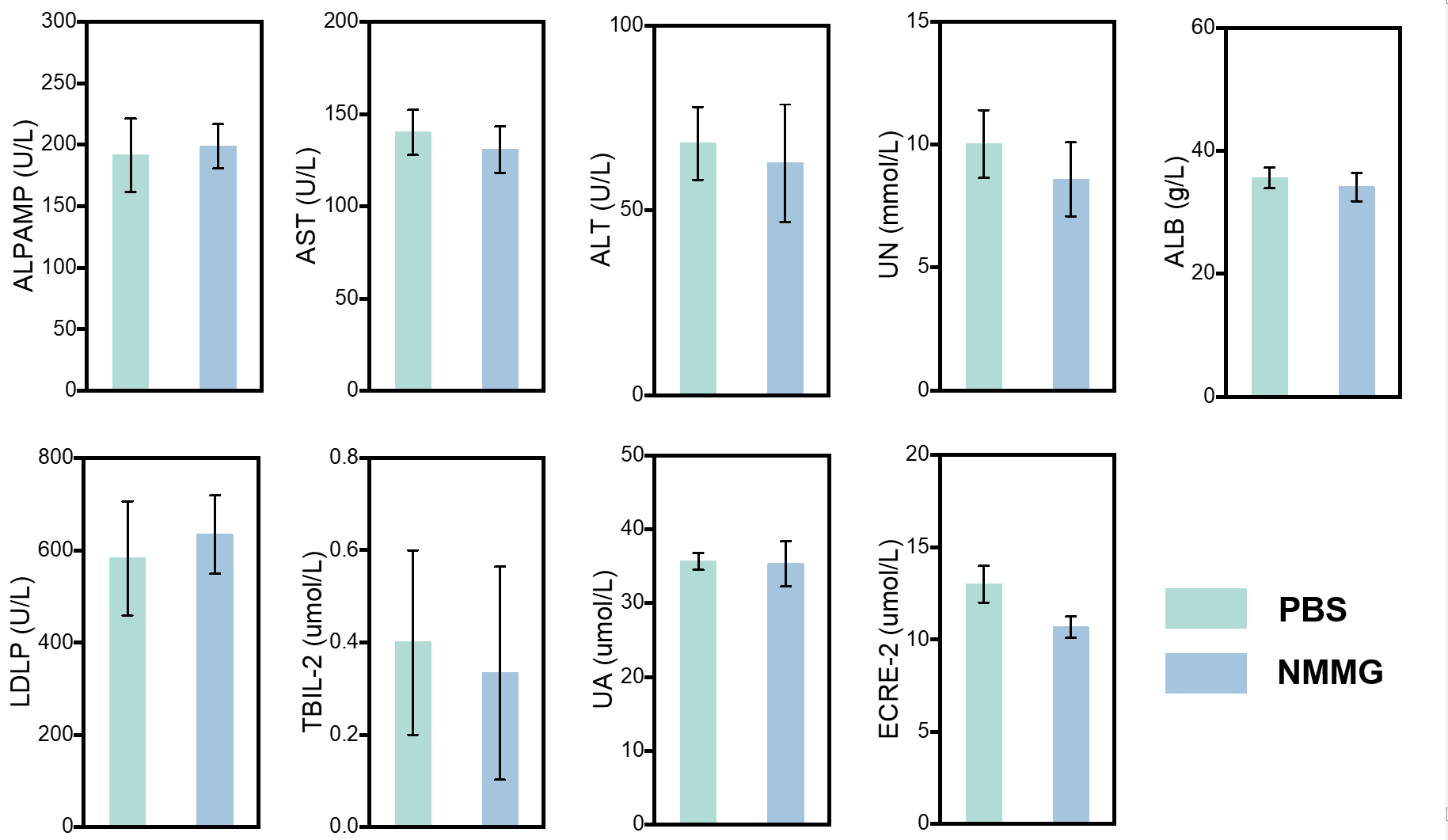


**Figure S16.** The hematological analysis after NMMG treated (n=5).


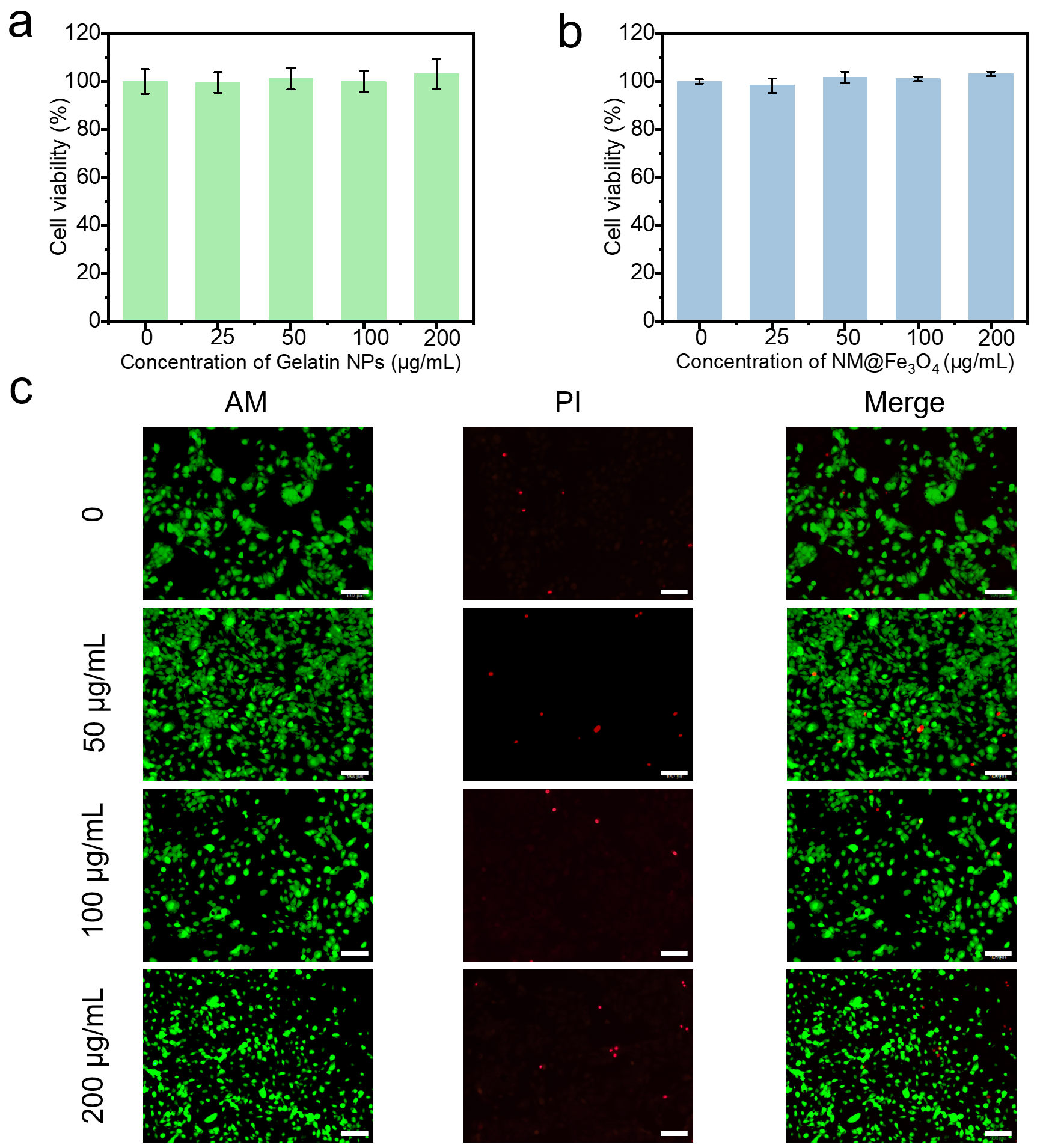


**Figure S17.** In vitro cell biocompatible evaluation. The cell viability of HaCaT cells incubated with various concentrations of (a) Gelatin NPs and (b) NM@Fe_3_O_4_. (c) Fluorescence photos of AM/PI double staining of dead and alive after co-incubating cells with NMMG at different concentrations for 24 h. Scale bar: 100 μm.


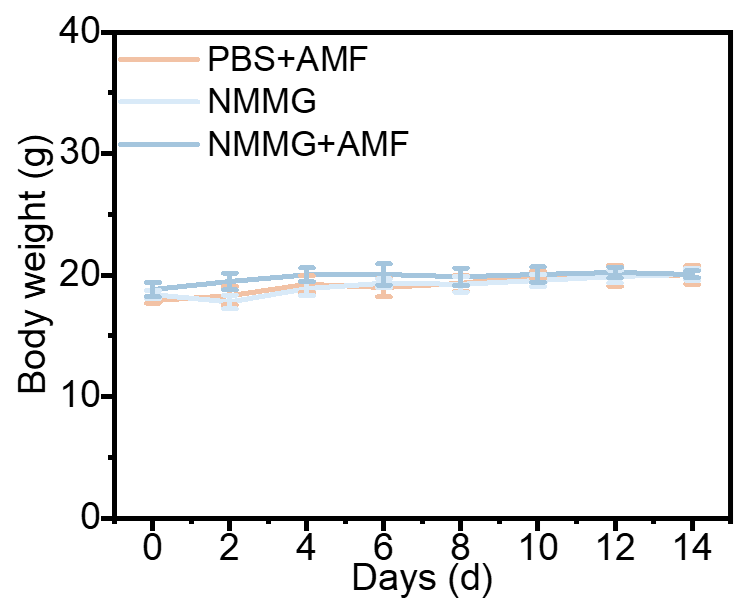


**Figure S18.** Body weight changes of all groups during the treatments (n=5).


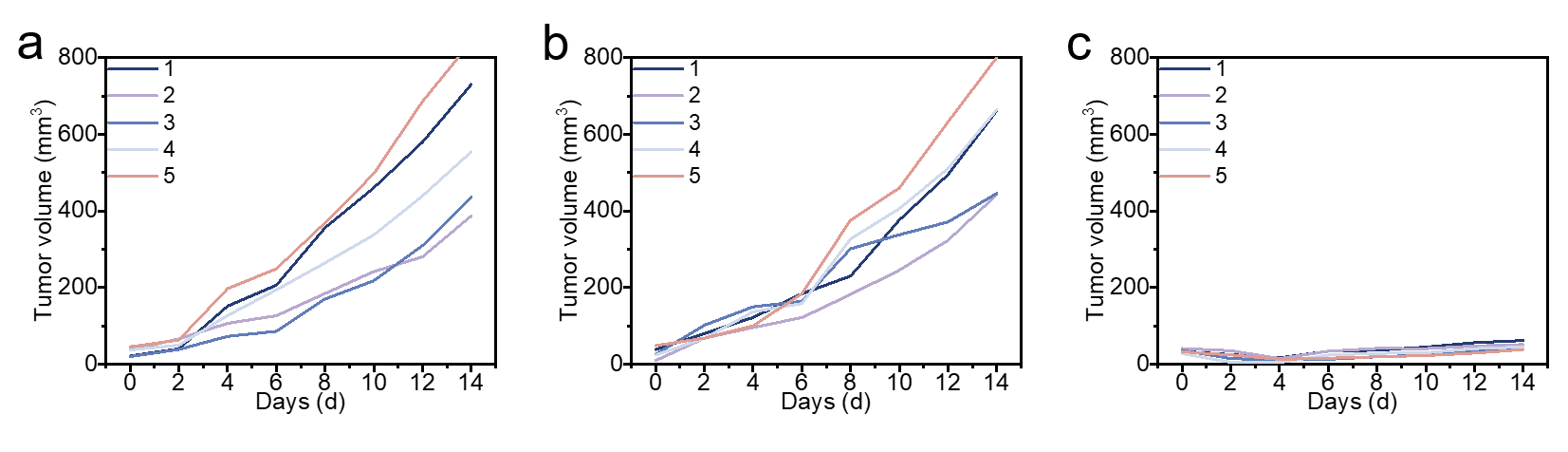


**Figure S19.** The tumor growth curves of each mouse in different treatment groups (n=5): (a) PBS, (b) NMMG, (c) NMMG+AMF.


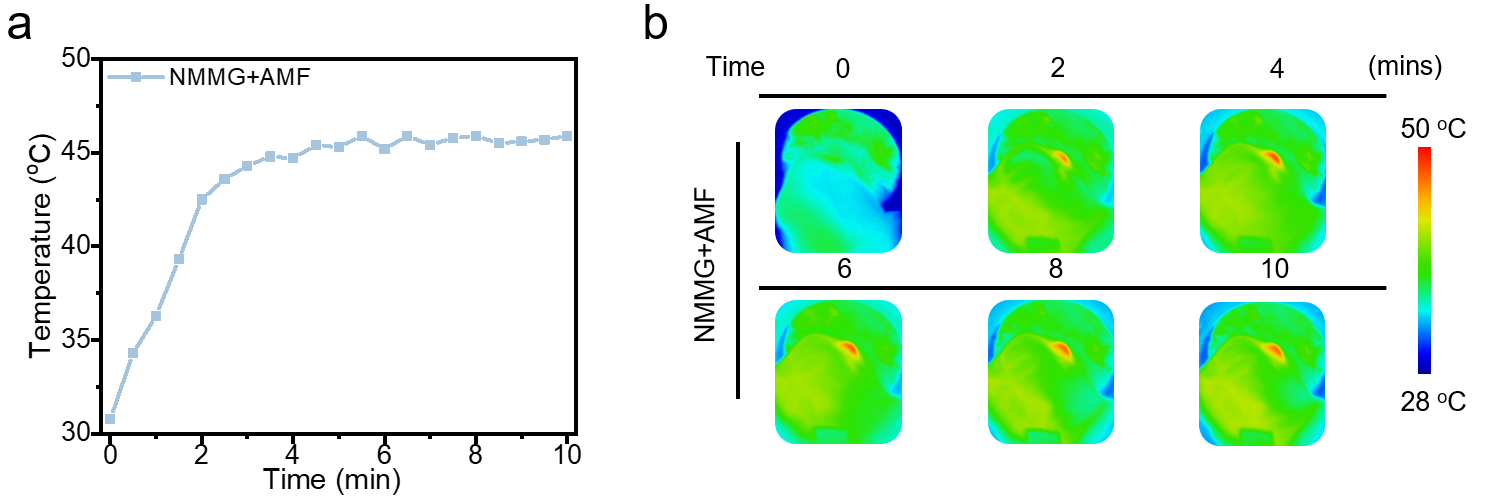


**Figure S20.** (a) Temperature curves and (b) infrared thermal images of the embolized rabbit ears with NMMG under AMF.


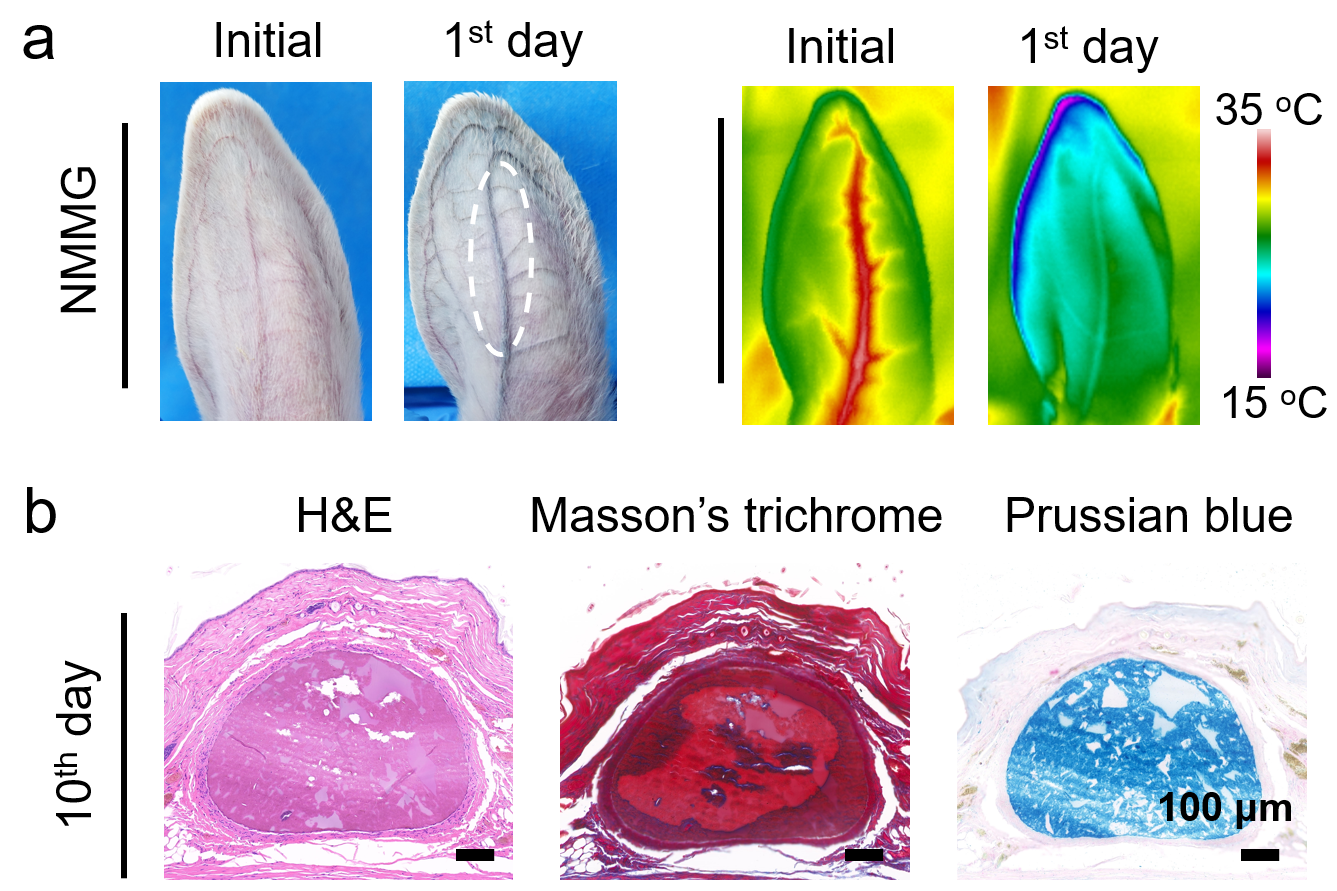


**Figure S21**. (a) Digital photographs and near-infrared thermal images of rabbit ears before and after embolization with NMMG. (b) H&E, Masson’s trichrome, and Prussian blue staining of cross-sections of the embolized rabbit ear artery at day 10 post-embolization (scale bar: 100 μm).


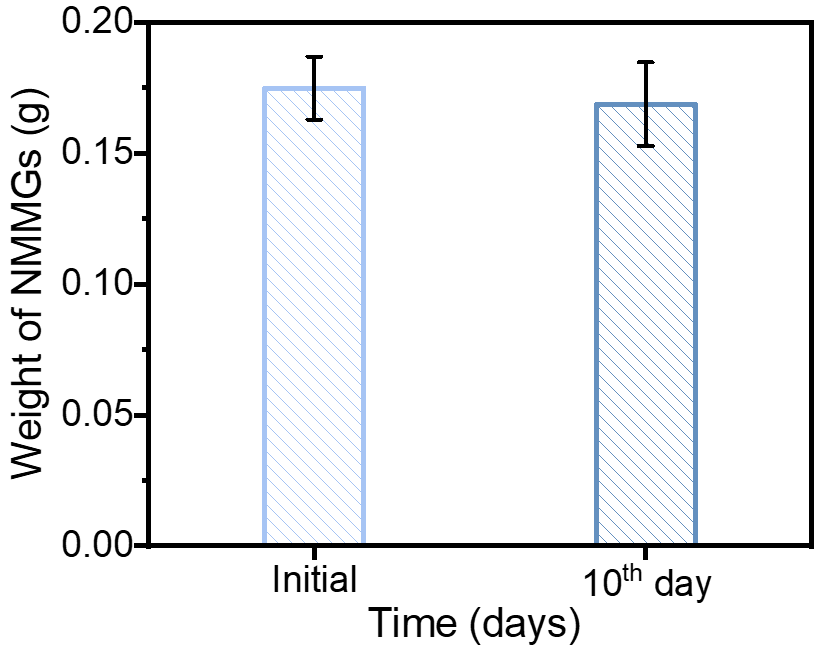


**Figure S22**. Comparison of NMMGs weight before implantation and after 10 days in vivo (n=3).

**Supplementary References**

1. X. F. Pan, H. L. Gao, Y. Lu, C. Y. Wu, Y. D. Wu, X. Y. Wang, Z. Q. Pan, L. Dong, Y. H. Song, H. P. Cong, S. H. Yu, *Nat. Commun.* **2018**, *9*, 2974.
2. A. Senthilnathan, D. M. S. N. Dissanayake, G. T. D. Chandrakumara, M. M. M. G. P. G. Mantilaka, R. M. G. Rajapakse, H. M. T. G. A. Pitawala, K. M. Nalin de Silva, *R. Soc. Open Sci*. **2019**, *6*, 182212.
3. W. Chen, S. Li, C. Chen, L. Yan, *Adv. Mater*. **2011**, *23*, 5679.
4. H. Y. Yang, H. Wang, C. H. Wen, S. Bai, P. F. Wei, B. Xu, Y. J. Xu, C. Z. Liang, Y. J. Zhang, G. L. Zhang, H. Q. Wen, L. Zhang, *J. Nanobiotechnology* **2022**, *20*, 98.
